# Charge transport in individual short base stacked single-stranded RNA molecules

**DOI:** 10.1038/s41598-023-46263-0

**Published:** 2023-11-13

**Authors:** Subrata Chandra, Ajoke Williams, Farkhad Maksudov, Evgenii Kliuchnikov, Keshani G. G. Pattiya Arachchillage, Patrick Piscitelli, Aderlyn Castillo, Kenneth A. Marx, Valeri Barsegov, Juan M. Artes Vivancos

**Affiliations:** Department of Chemistry, University of Massachusetts, Lowell, 01854 USA

**Keywords:** Biophysics, Molecular electronics, DNA, RNA

## Abstract

Charge transport in biomolecules is crucial for many biological and technological applications, including biomolecular electronics devices and biosensors. RNA has become the focus of research because of its importance in biomedicine, but its charge transport properties are not well understood. Here, we use the Scanning Tunneling Microscopy-assisted molecular break junction method to measure the electrical conductance of particular 5-base and 10-base single-stranded (ss) RNA sequences capable of base stacking. These ssRNA sequences show single-molecule conductance values around $10^{-3}G_0$ ($G_0= 2e^2/h$), while equivalent-length ssDNAs result in featureless conductance histograms. Circular dichroism (CD) spectra and MD simulations reveal the existence of extended ssRNA conformations versus folded ssDNA conformations, consistent with their different electrical behaviors. Computational molecular modeling and Machine Learning-assisted interpretation of CD data helped us to disentangle the structural and electronic factors underlying CT, thus explaining the observed electrical behavior differences. RNA with a measurable conductance corresponds to sequences with overall extended base-stacking stabilized conformations characterized by lower HOMO energy levels delocalized over a base-stacking mediating CT pathway. In contrast, DNA and a control RNA sequence without significant base-stacking tend to form closed structures and thus are incapable of efficient CT.

## Introduction

Charge transport (CT) in biomolecules is pivotal for a range of biological processes^1–5^. In the particular case of oligonucleotides, RNA has become the focus of intensive biophysical and biochemical research, because RNA is the main biomolecule mediating how genetic information is translated to proteins in all cells and carries the genetic information in many pathogens, between several other functions^6,7^. Exploration of the CT process in oligonucleotides is crucial to the fundamental understanding of many biological mechanisms, enabling new nanotechnology applications based on nucleic acid nanostructures^8^, and designing next-generation single-molecule electrical biosensors^9^. In DNA, there is consensus on the notion that CT occurs via the delocalized Highest Occupied Molecular Orbital (HOMO) of the molecules, with competing tunneling and hopping mechanisms^10^. For short sequences, tunneling dominates, and in longer sequences, hopping through accessible HOMO orbitals becomes more important; intermediate and alternative mechanisms are also described in the literature^10,11^. The fact that both the conformation of oligonucleotide helices and the presence of base mismatches modulate the CT process also points to the fact that HOMO delocalized over several bases plays a major role in the CT process^12–14^. In contrast, recent reports on long DNA molecules in ultrahigh vacuum and low 5–60 K temperature^15^ suggest that the backbone of the oligonucleotide mediates CT in DNA. It is likely that both scenarios play some role in the nucleic acids CT general picture, probably at different energies (low energies mainly favoring through the base stacking, while high energies could open the backbone CT channels), but more research is needed to advance this knowledge. A recent study explores the conductance of thiolated and amino ssDNA and dsDNA; the conductance is almost double after hybridization^16^. Our ongoing studies on RNA and DNA:RNA hybrids show that these processes can differ in particular oligonucleotides too^9,17^. Taken together, these published reports show that more research is necessary to integrate all the experimental findings into a general model of CT in oligonucleotides. Recently, we reported the results of the first CT measurements on double-stranded RNA in which we compared the single-molecule electrical conductance of a dsRNA sequence with that for the same DNA:RNA hybrid sequence^17^. The average conductance values were found to be similar for both molecules, but the conductance of dsRNA duplex was more variable. This points to RNA exhibiting unexpected electronic behavior compared to that of DNA, and shows the need to characterize different kinds of oligonucleotides to explore the underlying CT mechanisms, in order to complete the physical picture behind their single-molecule electrical behaviors.

Here, we investigated the CT characteristics of single-stranded RNA oligonucleotides and compared them with their DNA counterparts at the single-molecule level using the Scanning Tunneling Microscopy-assisted Molecular Break Junction (STM-BJ) approach. We find a molecular conductance peak for base stacked short ssRNAs, while ssDNA results in featureless conductance histograms. To understand the structural features responsible for the measured differences, we carried out CD studies of the RNA and DNA oligonucleotides and employed Molecular Dynamics (MD) simulations and Machine Learning to identify the specific oligonucleotide conformers and calculate their equilibrium populations that optimally reconstituted the CD spectra. We then used these specific conformers in Density Functional Theory calculations to interpret the single-molecule experimental results. In contrast to the equivalent ssDNA oligonucleotides, for ssRNA conformers, we found preferentially extended conformations reinforced by base stacking, which are characterized by lower-energy HOMO orbitals partially delocalized over the molecular structure. Thus, the different factors necessary for efficient CT in these single-stranded oligonucleotides are, (i) extended conformations leaving the molecular ends accessible for molecular binding with electrodes and (ii) energetically accessible molecular orbitals mediating the transport resulting from the delocalization over a base-stacked CT pathway.

## Results and discussion

**Figure 1:**
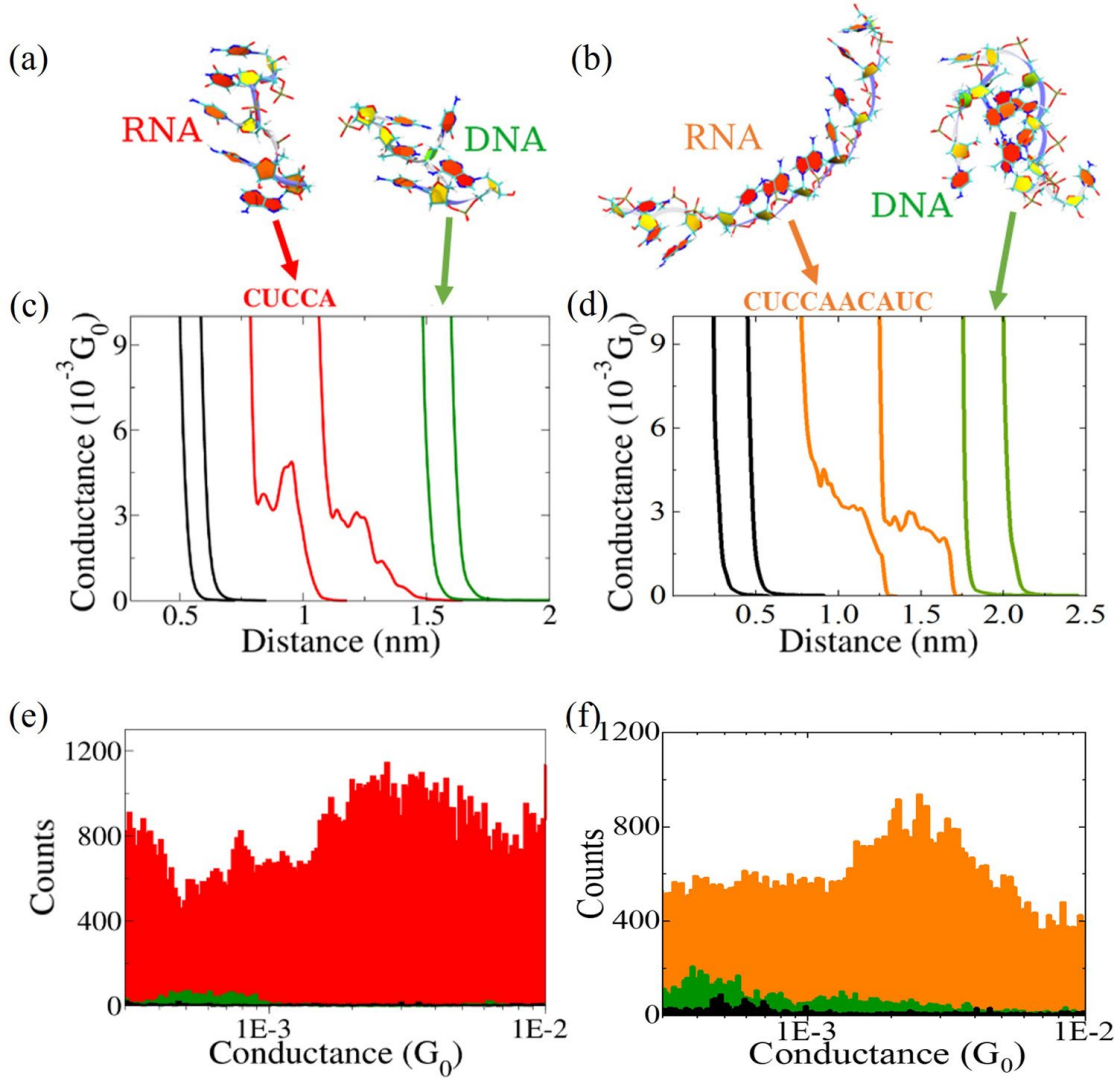
Single-molecule electrical measurements of single-stranded oligonucleotide sequences: Molecular Dynamics snapshots of the most representative conformations for (**a**) 5-mer RNA and DNA and (**b**) 10-mer RNA and DNA shown in Twister representation (blue line going through the backbone) and in PaperChain representation (for nucleic bases). All the rings are colored by pucker, using the Cremer-Pople pucker amplitude^35^. (**c**) Conductance vs. distance curve examples (black for phosphate buffer blank, red for ssRNA, and green for ssDNA) for 5-mer sequences. (**d**) Example raw data curves for 10-mer sequences (black for buffer, orange for RNA, green for DNA). (**e**) Logarithmic conductance histogram for 5-mer RNA and (**f**) 10-mer RNA showing the average conductance around 3 × $10^{-3}G_0$ ($G_0= 2e^2/h = ~7.75\times 10^{-5}$ A/V is the quantum of conductance). The background signals from a control phosphate buffer experiment are shown in black, RNA data are in red and orange, and DNA signals are in green. Experiments were performed with a constant 20 mV applied bias. Histograms include around 300 curves from a total of 5000.

We explored single-stranded (ss) oligonucleotides of two different lengths: five bases (5-mer), and ten bases (10-mer). Preliminary Molecular Dynamics (MD) simulations revealed the preponderance of base-stacked extended conformation for 5-mer ssRNA (sequence CUCCA) as compared with the collapsed structure for the equivalent 5-mer ssDNA (sequence CTCCA; Fig. 1a). Similarly, the 10-mer ssRNA (CUCCAACAUC) shows an extended form with bases stacked for most of the molecular length, whereas the 10-mer equivalent ssDNA (right, CTCCAACATC) displays a collapsed state without significant base stacking (Fig. 1b). These results of computational molecular modeling suggest that, if the CT process occurs mainly through the HOMO orbitals of the stacked bases, then the 5-mer and 10-mer ssRNA molecules should be able to support CT. Motivated by these MD observations we explored the electrical conductance of the 5-mer and 10-mer ssRNA and ssDNA oligonucleotides. These studies constitute the first conductance measurements on single-molecule ssRNA. Figure 1c shows an example of conductance vs. distance (red) traces for a base-stacked 3’ and 5’ thiolated 5-mer ssRNA, which show steps or plateaus around $3\times 10^{-3}G_0$, where $G_0= 2e^2/h = ~7.75\times 10^{-5}$ A/V(S) is the quantum of conductance (*e* is the electron charge and *h* is the Planck constant). In contrast, the control experiments on phosphate buffer solution without RNA (black curves) and experiments on the equivalent non-base-stacked 5-mer ssDNA (green curves) showed the exponential decaying curves without any features (neither step(s) nor plateau(s); Fig. 1c). We obtained similar results for the 10-mer ssRNA (orange) and ssDNA oligonucleotides (Fig. 1d). Gathering thousands of the conductance vs. distance traces and using automated algorithms^18^ enabled us to construct conductance histograms for 5-mer and for 10-mer oligonucleotides. By fitting the Gaussian distribution to the histogram peaks, we obtained the most probable conductance values, which came to $3.6\pm 0.2 {\times 10^{-3}}G_0$ and $2.9\pm 0.4 {\times 10^{-3}}G_0$ for 5-mer and 10-mer ssRNA oligonucleotides, respectively. In Supplementary Fig. S1 there are expanded single-molecule conductance results from these experiments, showing 2D histograms with all the included curves with equivalent results.

In an alternative approach, we used the current-time method to measure the spontaneous formation of RNA molecular junctions^19,20^. In the current-time method, we bring the tip to tunneling distance from the substrate, then the STM feedback is turned off while the current is being measured as a function of time. When a molecule spontaneously binds between the two electrodes, a sudden jump or ”blink” is recorded in the current-time trace (see SI Figs. S2b,c). Figures S2d and e show the conductance histograms for the above-mentioned traces showing peaks for 5-mer and 10-mer ss RNA sequences respectively. The most probable conductance value obtained from cumulative histograms (Figs. S2f and g) with this technique match those obtained with the STM-BJ method discussed above, indicating that the forces applied by the STM electrodes in junction formation do not affect the molecular conformations of the oligonucleotides studied. Interestingly, the conductance histograms for the equivalent 5-mer and 10-mer ssDNA sequences did not show any features in this conductance range from $10^{-4}G_0$ to $10^{-2}G_0$ (green histograms in Fig. 1). In additional control experiments on a different ssRNA of the same length but not capable of base stacking (sequence CUCCACUCCA), the conductance histogram did not show any peaks (see Fig. S3), indicating that molecular conductivity is exclusive for these particular ssRNA sequences probably having base stacking and an open conformation.

**Figure 2:**
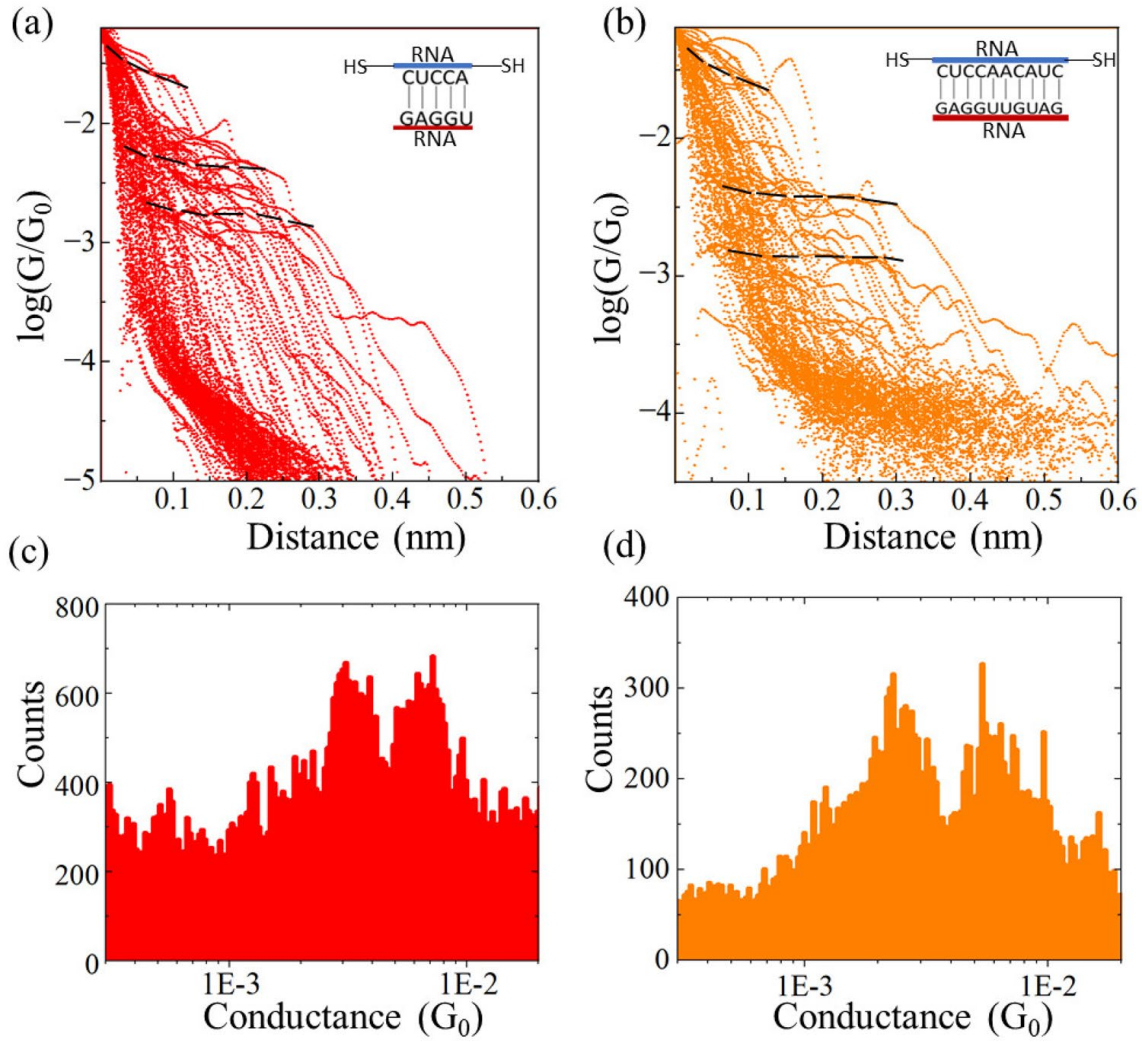
Conductance measurements of thermally hybridized dsRNA sequences: Experimental 2D logarithmic conductance–distance (log(G/$G_0$)– distance) traces for (**a**) 5-mer dsRNA and (**b**) 10-mer dsRNA with conductance step around two different conductance values, i.e. at 3 × $10^{-3}G_0$ and 6 × $10^{-3}G_0$ ($G_0= 2e^2/h = ~7.75\times 10^{-5}$ A/V is the quantum of conductance), master curves (indicating most probable conductance) are denoted by black dashed lines. (**c**) 1D conductance histogram for 5-mer dsRNA duplex and (**d**) for 10-mer dsRNA duplex, both showing bimodal distribution matches with the 2D results. Experiments were performed in 100 mM phosphate buffer with a constant 20 mV bias. The histograms include around 250 curves out of a total of 5000 per dataset.

To gather more insights about the electronic properties of the RNA molecules described above, we compared their electronic properties with those of their corresponding double-stranded (ds)RNA duplexes. We hybridized the ssRNA strands with their complementary strands by adding both strands in solution, heating this solution up to 80°C and cooling it down to room temperature. Next, we carried out the STM-BJ based electrical conductance measurements. The conductance histograms for the 5-base pair (bp) and 10-bp dsRNA duplexes are displayed in Fig. 2a and b, which show reproducible conductance steps that form around two different conductance values ($3\times {10^{-3}}$ $G_0$ and $6\times {10^{-3}}$ $G_0$). By inspecting the conductance histograms (Fig. 2c,d), we notice that one of the peaks directly matches the conductance peak for the ssRNA (compare the histograms in Figs. 1 and 2), while the other peak is a new peak that appears in a higher conductance range. This second peak value (around $6\times {10^{-3}}$ $G_0$) probably reflects a more stable base stacking in dsRNA (a better and more stable CT pathway), while the low conductance peak indicates that a significant amount of ssRNA that has not formed a double helix but remains in single-stranded form. This behavior is not surprising for short sequences, such as the 5-bp and 10-bp dsRNA duplexes studied. Indeed, we estimated the melting temperature ($T_m$) for these duplexes, and we found that $T_m =9^{\circ }$C and 23°C for the 5-bp and 10-bp dsRNAs, respectively (see melting temperature calculation section in SI for details). Hence, at room temperature, a significant fraction of the total population of ssRNA molecules remains single-stranded. Note that experiments on the target RNA sequences without thiol binding groups (complementary to the sequences studied in Fig. 1) result in featureless histograms, essentially equivalent to a blank experiment performed in the absence of biomolecules in solution (compare the histograms in the SI Fig. S4).

**Figure 3:**
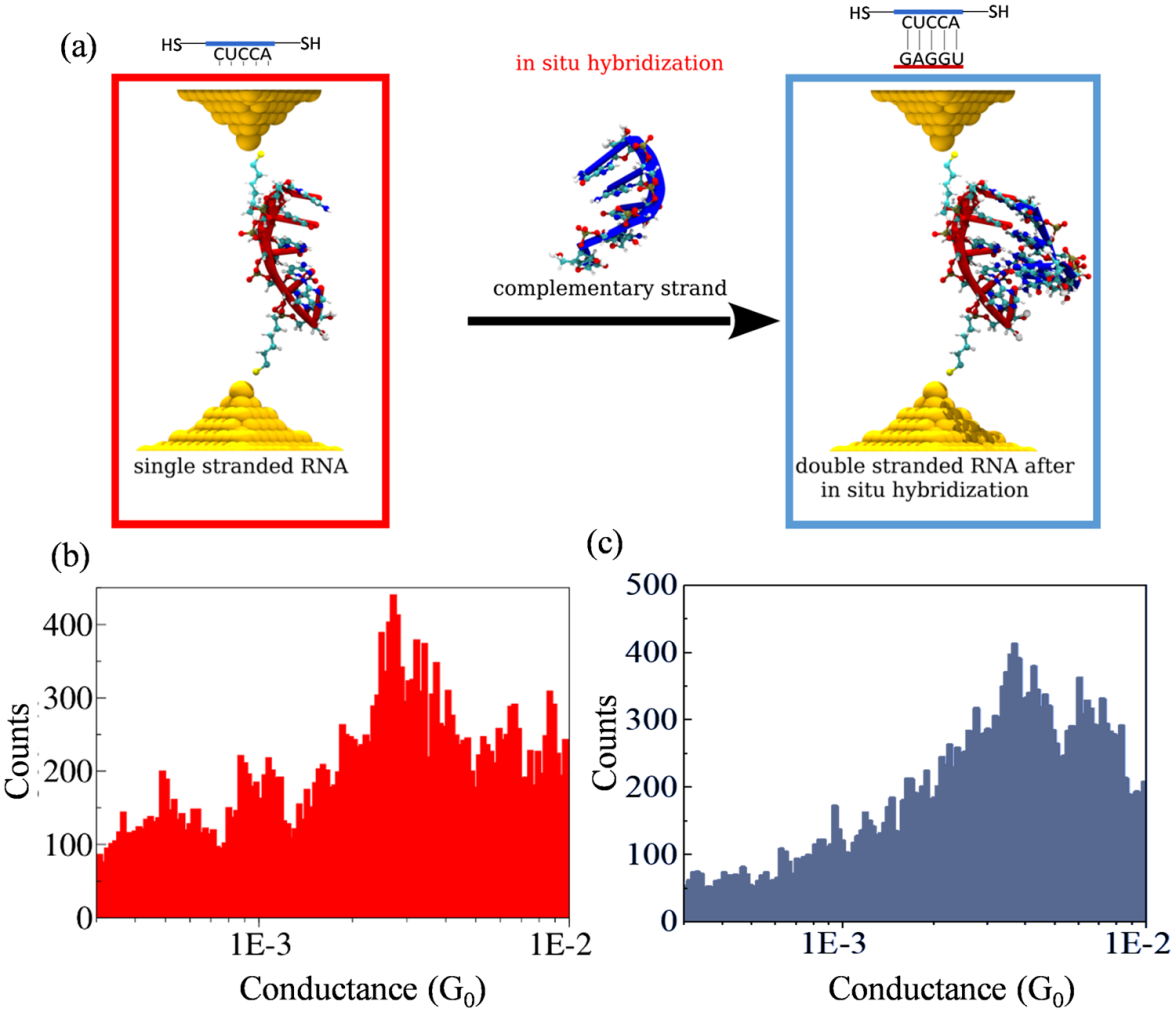
Conductance measurements of in-situ hybridized dsRNA (5 mer) sequence CUCCA (**a**) Schematic representation of the experiment. Gold electrodes are depicted using yellow balls. Each RNA strand displays the secondary structure and chemical bonds with “NewCartoon” and Bonds representations implemented in VMD^35^. Logarithmic conductance–distance (log(G/$G_0$) – distance) histograms for (**b**) single-stranded 5-mer RNA before and (**c**) 5-mer RNA after *in situ* hybridization with the complementary sequence indicated in a, showing two peaks. $G_0= 2e^2/h = ~7.75\times 10^{-5}$ A/V is the quantum of conductance. Experiments were performed in 100 mM phosphate buffer with a constant 20 mV bias. The histograms include around 250 curves out of a total of 5000 per dataset.

To test this hypothesis about the single-stranded and double-stranded forms of RNA coexisting, we performed in situ hybridization experiments (graphically illustrated in Fig. 3a). First, we measured the conductance of the thiol-modified ssRNA sequence. Next, we added the complementary strand at room temperature and then acquired more conductance curves after a 30-min incubation period. The initial conductance histograms (Figs. 3b) show single conductance peaks in agreement with the conduction peaks shown in Fig. 1 for 5-bp ssRNA sequences. Upon the addition of the complementary strands, the conductance curves now show plateaus at two different conductance values (Fig. 3c), resulting in two separate conductance peaks in the conductance histograms, which match the conductance peaks of the histograms in Fig. 2. For the 10-bp ssRNA sequence also, on the addition of the complementary strand at room temperature, we observed two separate conductance peaks shown in Fig. S5 in SI. This result supports our hypothesis that both single-stranded and double-stranded forms of 5-mer and 10-mer RNAs coexist at equilibrium. These single-molecule conductance results are consistent with several reported results for ds oligonucleotides of similar lengths in the literature^14,21–23^. Note that these sequences only have partial base stacking and thus result in a modest length dependence for the conductance, indicating that the crucial factor for CT in oligonucleotides are the stacked bases (more than the molecular length in itself). For both of these short RNA sequences, the conductance values obtained for double-stranded RNA contain a significant contribution from the single-stranded population of molecules, thus giving rise to two different conductance peaks (residual conductance peaks observed in the high ${10^{-2}}$ $G_0$ range are the result of transient trapping of water molecules at the junctions, as recently described^24^).

**Figure 4:**
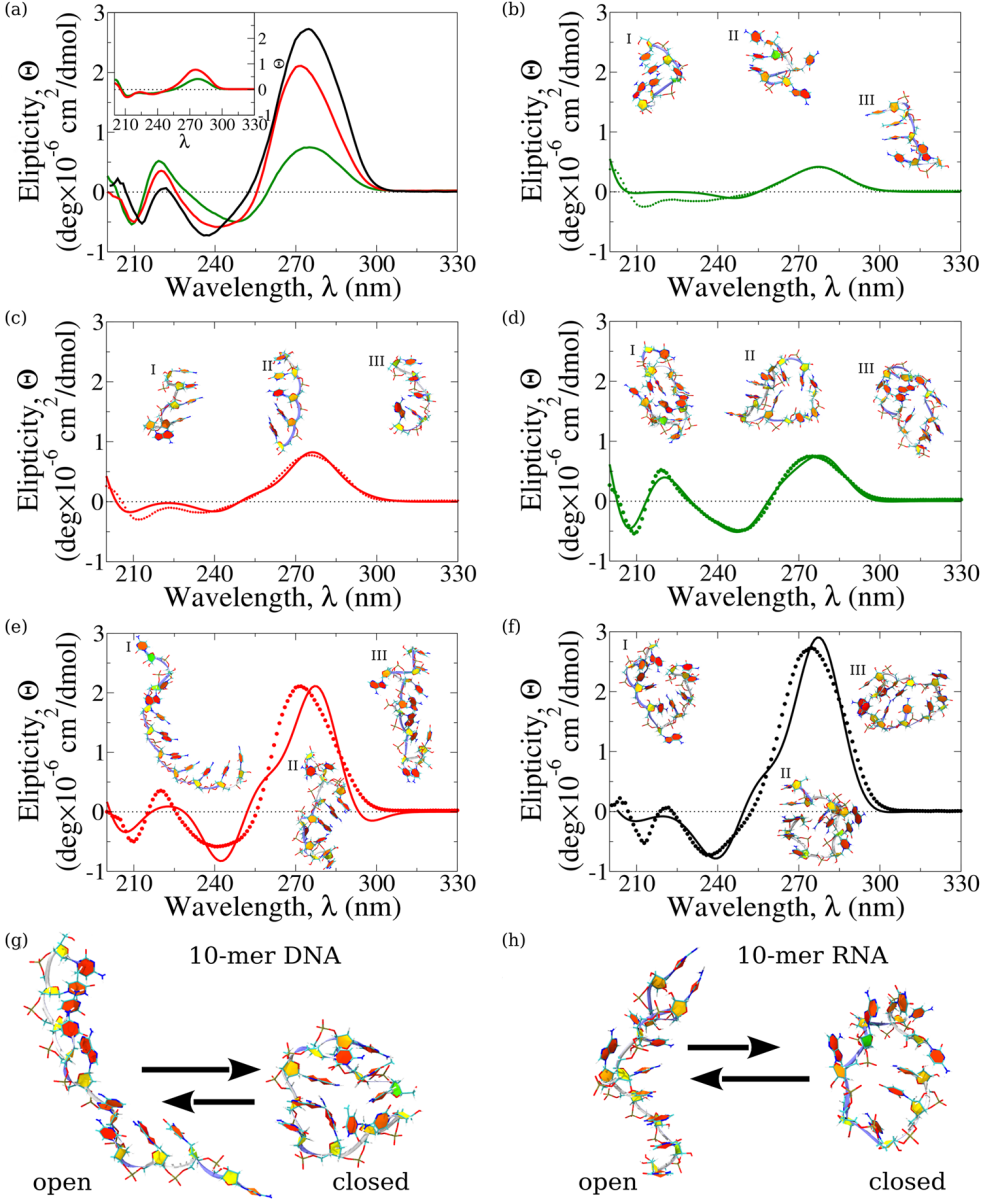
Circular dichroism spectra of single stranded oligonucleotide sequences: (**a**) Experimental CD spectra for single-stranded 10-mer RNA (red curve) and 10-mer DNA (green curve), and for control (non base-stacking) 10-mer RNA (black curve). *The inset* shows CD spectra for the single-stranded 5-mer RNA (red curve) and 5-mer DNA (green curve). (**b**)–(**f**) Superposed are the calculated average CD profiles for the single-stranded 5-mer DNA (panel (**b**)), 5-mer RNA (panel (**c**)), 10-mer DNA (panel (**d**)), 10-mer RNA (panel (**e**)) and control 10-mer RNA (panel (**f**)), obtained experimentally (dotted lines) and theoretically (solid lines). The structure snapshots, numbered I, II, and III, correspond to the first three most representative, highest weight solution conformations contributing to the ensemble average CD spectra. Also shown are representative examples of extended conformations (’open’ state) and collapsed conformations (’closed’ state) for the 10-mer DNA (panel (**g**)) and 10-mer RNA (panel (**h**)). Arrows indicate the reversible transition between these two states. Structure snapshots in panels (**b**)–(**h**) are shown in Twister representation (blue line running through backbone) and in PaperChain representation (for nucleic bases). All the rings are colored by pucker, using the Cremer–Pople pucker amplitude^35^.

To better understand the underlying structural characteristics that result in the differences in CT behavior observed in base-stacked short single-stranded oligonucleotides vs. non-base-stacked oligonucleotides , we performed Circular Dichroism (CD) experiments. CD spectroscopy is a valuable tool to map the chiral conformational properties of biomolecules^25^ that carry information about their secondary structures, which in the case of oligonucleotide sequences depends on the stacking and orientation of the bases. CD spectra for the 5-mer and 10-mer ssRNA and ssDNA oligonucleotides in Fig. 4 show that, although the shapes of the CD spectra look similar, there is a significantly larger differential absorbance in the dominant positive band near the wavelength $\lambda$ = 270 nm for ssRNA compared to ssDNA. For 5-mer ssRNA , the ellipticity at $\lambda$ = 270 nm is nearly twice that of 5-mer ssDNA , while for 10-mer this dominant positive band is about four times higher (Fig. 4a). A higher peak intensity around $\lambda$ = 270 nm corresponds to a more A-form-like type right-handed helical base orientation in the oligonucleotide conformation, where the bases are stacked parallel to each other and not perpendicular to the helix axis (as in the B-form type canonical dsDNA)^25^. For 5-mer and 10-mer ssDNA, the lower peak height corresponds to a lower extent of the right-handed base stacking conformation, resulting in lower overall chirality. Additional structural possibilities can be a loop-shaped structure or a ”closed” form in this ssDNA^25^.

These CD observations correlate well with our findings from the single-molecule conductance measurements about a more efficient electrical conductance in ssRNA, as compared with ssDNA, where favorable CT pathways exist via the electronic orbitals of the stacked bases reinforcing the ’open’ extended conformations (in 5-mer and 10-mer ssRNAs), whereas the ’closed’ conformations (in 5-mer and 10-mer ssDNA) might not present such pathways and might not be capable of forming stable junctions with the electrodes. To investigate these possibilities and to better understand the structural rules underlying the observed differences in the measured ssRNA vs. ssDNA conductance values, we turned to computational modeling of the molecular structures and theoretical exploration of the electronic properties of the ssRNA and ssDNA sequences described below.

**Figure 5:**
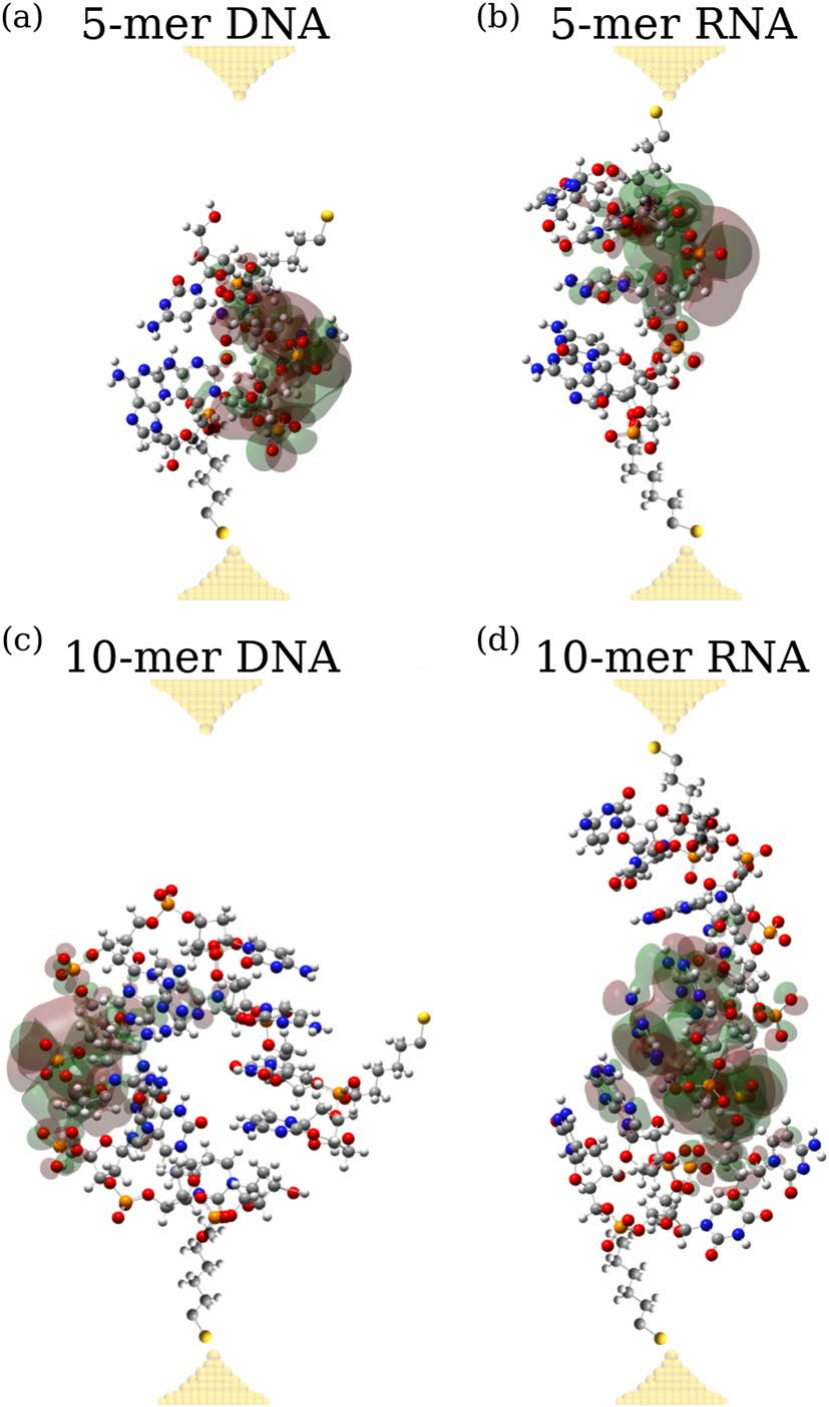
Molecular orbitals for the HOMO for single-stranded RNA and DNA sequences: The MD snapshots superposed with their HOMO orbitals for the average structures of collapsed 5-mer DNA (panel (**a**)), extended 5-mer RNA (panel (**b**)), collapsed 10-mer DNA (panel (**c**)), and extended 10-mer RNA (panel (**d**)) obtained from the DFT-based quantum chemistry calculations, which are based on the average structures of RNA and DNA sequences identified in the all-atrom MD simulations. The RNA and DNA molecules are displayed in atomic representation (carbon, oxygen, nitrogen, and hydrogen atoms are shown in grey, red, blue, and white colors respectively). The sulfur atoms of the thiol linkers are shown in gold color. Green and red colors represent negative and positive energy iso-surfaces of HOMO, respectively (iso-value = 5 × $10^{-4}$ $e/a_{0}^{3}$, where the Bohr radius $a_{0}$ = 0.529 Å). Gold electrodes are shown by semi-transparent yellow balls.

We explored the electronic properties of 5-mer and 10-mer oligonucleotides. We combined the all-atom MD simulations with the DFT-based quantum chemistry calculations to visualize the electronic densities associated with the ’open’ and ’closed’ oligonucleotide conformations discussed above. First, for each single-stranded oligonucleotide (5-mer and 10-mer ssRNAs and ssDNAs) and the control oligonucleotide (10-mer ssRNA), we generated 10 independent 2-µs long MD simulation runs (total time 20 µs; see “Methods” and Fig. 5). Interestingly, the results obtained revealed distinctly different conformational dynamics for the single-stranded DNA and RNA molecules. Here, we describe our results for 10-mer ssDNA and ssRNA, and for control 10-mer ssRNA. For ssDNA sequences, the overwhelming majority of structure snapshots showed the ssDNA molecules adopting the collapsed shape (i.e. ’closed’ state), in which the 5'- and 3'-ends of the molecule are close to each other; a few snapshots displayed the ssDNA molecules in the extended shape (i.e. ’open’ state), in which the 5'- and 3'-ends are far apart (Fig. 4d; see also Supplementary Movie S1). For example, the populations of the closed and open conformations for 10-mer ssDNA came to 64% and 36%, respectively. By contrast, the conformational dynamics for single-stranded RNA sequences showed the prevalence of extended conformations (Fig. 4e; see also Supplementary Movie S2) with the 5'- and 3'-ends far apart. For example, the population of closed and open conformations for 10-mer ssRNA were 45% and 55%, respectively. The control 10-mer ssRNA sequence (CUCCACUCCA) was found to populate the collapsed conformations as well as the extended conformations (see Supplementary Movie S3), and the population of closed and open conformations came to 55% and 45%, respectively. Even if these differences may seem small, they show a substantial trend about the two extreme conformations for these molecules; base-stacked sequences can bind the electrodes and provide a measurable conductance (ssRNA) while non-stacked oligos (ssDNA and the control ssRNA) tend to be closed and either don’t form the junction and/or cannot support efficient CT. These results indicate (i) that single-stranded RNA molecules exist predominantly in the extended conformations, thus, populating most of the time the ’open’ state, characterized by a large number of base stacks; and (ii) that the extent to which these ssRNA sequences populate the extended conformations also depends strongly on RNA sequence composition and internal base stacking .

Next, using these results from the all-atom MD simulations, we extracted the average structures of 10-mer ssDNA in the closed state, 10-mer ssRNA in the open state and control 10-mer ssRNA in the closed state (see “Methods”). These conformations were then used in the DFT-based quantum chemistry calculations to illuminate their electronic structures displayed in Fig. 5. The HOMOs are compared for 5-mer ssDNA and 5-mer ssRNA are displayed in Fig. 5a and b, respectively, and for 10-mer ssDNA and 10-mer ssRNA in Fig. 5c and d, respectively. We see that the collapsed conformations of 5-mer and 10-mer ssDNA are probably not accessible to the electrodes, and do not form a molecular junction, hence resulting in featureless histograms (Fig. 5a and c). By contrast, the more extended conformations of both 5-mer ssRNA and 10-mer ssRNA allow both for the formation of proper molecular attachments with the electrodes and for delocalized HOMOs over a base-stacked pathway (Fig. 5b and d). Indeed, we estimated the distance between the thiol groups (end-to-end distance *X*) and the degree of HOMO delocalization for these oligonucleotides. We found that the end-to-end distance is *X* = 21 Å and 26 Å for 5-mer ssDNA and 5-mer ssRNA, respectively, and *X* = 28 Å and 36 Å for 10-mer ssDNA and 10-mer ssRNA , respectively. The degree of HOMO delocalization is 12Å and 13Å for 5-mer ssDNA and 5-mer ssRNA, respectively, and 13 Å and 15 Å for 10-mer ssDNA and 10-mer ssRNA, respectively. These results confirm our previous findings, namely that more extended conformations adopted by RNA molecules, which are reinforced by the base-stacking interactions, facilitate CT.

Due to rugged energy landscapes and the underlying conformational transitions in RNA and DNA molecules^26–29^, the brute-force MD simulations described above might not be able to resolve the most abundant molecular conformations. Here, we coupled the Circular Dichroism experiments with the MD simulations and Machine Learning assisted interpretation of the CD spectral lineshapes: (i) to resolve the entire ensemble of conformations for the 5-mer and 10-mer oligonucleotides coexisting at equilibrium, and (ii) to describe the most abundant oligonucleotide solution conformers.

Using the output from MD simulations for each oligonucleotide system (10-mer ssDNA, 10-mer ssRNA, and control 10-mer ssRNA) , we selected conformations characterized by large negative free energies (*G*) [which correspond to large negative enthalpies (*H*) and large positive entropies (*S*)]. As an example, the histograms of these thermodynamic state functions for 10-mer ssRNA are displayed in Fig. S7. For each of these conformations selected, we calculated the CD spectral lineshape $\theta _{i}(\lambda )$ (where $i = 1, 2, \ldots , N$—total number of conformations for each single-stranded oligonucleotide system). Different weighted superpositions of these lineshapes $\sum _{i}w_{i}\theta _{i}(\lambda )$ and non-linear regression was used to fit the (average) experimental CD spectrum $\Theta (\lambda )$ (see “Methods”). This helped us to resolve the most relevant solution conformer structures and to evaluate their statistical weights (populations) $w_{i}$, treated as regression coefficients. This approach was used in a recent publication^30^ to model CD spectra for phosphorodiamidate morpholino oligonucleotides. Also, a similar approach was successfully used in a previous study by some of the coauthors to model the Small Angle X-ray Scattering (SAXS) data^31^.

Figure 4 shows the fitting of the CD spectra for the 5-mer ssRNA, 5-mer ssDNA, and the 10-mer ssRNA, 10-mer ssDNA, and the non-conductive control 10-mer ssRNA, along with the first three most populated conformations I, II, and III. The five most populated conformations I–V for the 10-mer ssRNA, 10-mer ssDNA and control 10-mer ssRNA, which account for $\sim$ 85% (i.e. majority) of all solution conformations, are characterized in Table 1. We explored the dynamic structural properties of conformations of these three single-stranded oligonucleotides in an aqueous solution in terms of the end-to-end distance *X*, which describes the overall molecular shape, and the numbers of base stacks $N_{BS}$ and base pairs $N_{BP}$, which carry information about the secondary structures of these oligonucleotide sequences. We found that *X* varies between 1.5 nm and 3.7 nm for 10-mer ssRNA, between 1.0 nm and 1.8 nm for 10-mer ssDNA, and between 1.1 nm and 2.8 nm for control 10-mer ssRNA (Table 1). $N_{BS}$ varies between 2 and 5 for 10-mer ssRNA, between 1 and 6 for 10-mer ssDNA, and between 1 and 5 for 10-mer ssRNA. $N_{BP}$ varies between 0 and 1 for the 10-mer ssRNA, between 1 and 3 for 10-mer ssDNA, and between 0 and 4 for control 10-mer ssRNA (Table 1). The most striking difference between the weighted (ensemble) average values of these dynamics parameters is observed for the end-to-end distance *X*, which is $\overline{X}$= 2.79 nm ± 0.96 nm for 10-mer ssRNA, 1.49 nm ± 0.48 nm for 10-mer ssDNA and 1.48 nm ± 0.67 nm for control 10-mer ssRNA (Table 1). This stresses again the prevalence of extended conformations for the conducting 10-mer ssRNA. Also, ssRNA structures are characterized by a lower count of base pairs, but a higher count of base stacks (Table 1). For example, $\overline{N}_{BP}$ is smaller for 10-mer ssRNA (0.07) and larger for 10-mer ssDNA (2.26) and control 10-mer ssRNA (0.67); $\overline{N}_{BS}$ decreases from 3.7 for 10-mer ssRNA to 3.1 for 10-mer ssDNA. These results confirm that the main conformational reason for the large conductivity of 10-mer ssRNA and the absence of conductivity of its counterpart 10-mer ssDNA and the control 10-mer ssRNA is the extended conformation of the former (’open state’) versus the collapsed conformations of both 10-mer ssDNA and control 10-mer ssRNA (’closed’ state). This allows for efficient binding of the extended 10-mer ssRNA to the STM electrodes via the thiolated ends.

To gain a detailed energetic understanding of the differences in CT in solution structures of the oligonucleotide sequences studied, we performed DFT-based quantum chemistry calculations (see “Methods”) for the most populated solution structures identified above. We calculated the molecular orbitals and energies associated with the highest occupied molecular orbital (HOMO), $E_{HOMO}$, and the energy gap, $\Delta E = E_{LUMO} - E_{HOMO}$, between $E_{HOMO}$ and the energy associated with the lowest occupied molecular orbital (LUMO), $E_{LUMO}$.

**Figure 6:**
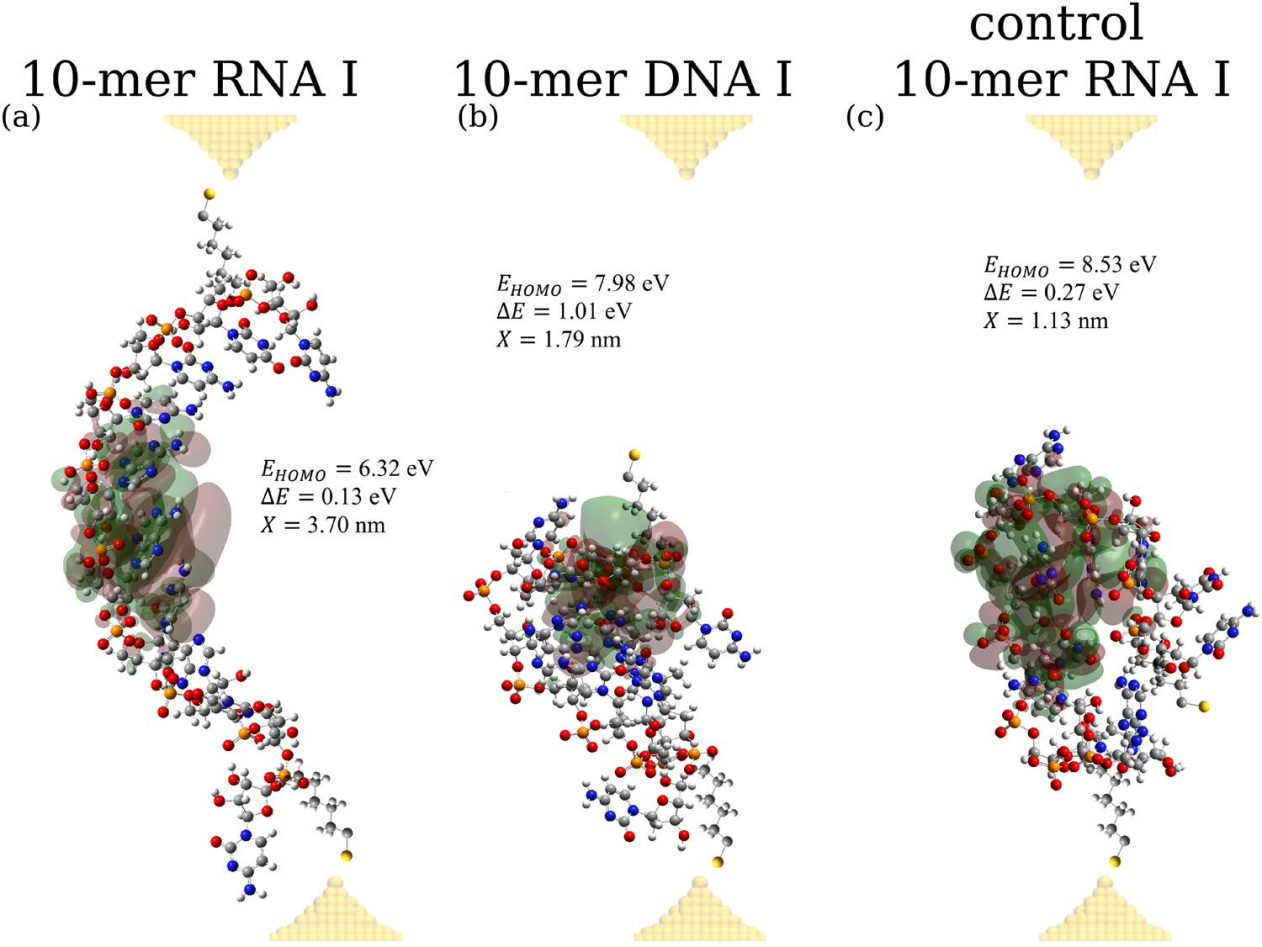
Molecular orbitals for the HOMO for the most populated conformations of single-stranded RNA and DNA sequences: Shown are the results of calculations for the full structures of 10-mer RNA (panel (**a**)), 10-mer DNA (panel (**b**)), and control 10-mer RNA (panel (**c**)) obtained with the DFT-based quantum chemistry calculations, which are based on the most abundant solution structures identified (see Table 1). The RNA and DNA molecules are displayed in atomic representation (carbon, oxygen, nitrogen, phosphorus and hydrogen atoms are shown in grey, red, blue, orange, and white colors respectively). The sulfur atoms of the thiol linkers are shown in gold color. Green and red colors represent negative and positive energy iso-surfaces of HOMO, respectively (iso-value = 5 × $10^{-4}$ $e/a_{0}^{3}$, where the Bohr radius $a_{0}$ = 0.529 Å). Gold electrodes are shown using semi-transparent yellow balls. The values of energy for the HOMO, $E_{HOMO}$ and the energy gaps, $\Delta E$, for these structures are displayed in the graph (see also Table 1).

The values of $E_{HOMO}$ for the first three most populated solution structures are accumulated and compared in Table 1. For solution structure I (most populated) of 10-mer ssRNA , $E_{HOMO}$ = 6.32 eV is the smallest, as compared to structure II with $E_{HOMO}$ = 7.47 eV and structure III with $E_{HOMO}$ = 7.11 eV, which is expected for the most populated conformation. The HOMO orbitals for the first most abundant structures for all three ten base length oligonucleotides are compared in Fig. 6, which shows that HOMO is delocalized the longest for 10-mer ssRNA (over 18 Å), as compared to 10-mer ssDNA (over 9 Å) and control 10-mer ssRNA (over 14 Å). 10-mer ssRNA in conformation I has the smallest energy $E_{HOMO}$ among all conformations for all oligonucleotides; for 10-mer ssDNA the smallest $E_{HOMO}$ was 7.88 eV and for control 10-mer ssRNA the smallest $E_{HOMO}$ was 7.30 eV. This is entirely consistent with the base-stacked 10-mer ssRNA being the only oligonucleotide capable of CT thanks to a lower energy HOMO orbital mediating the CT process. Similar results are obtained from the energies $E_{HOMO}$ for the second and third most populated solution conformations (see Table 1). The ensemble average HOMO energy, $\overline{E}_{HOMO}$, exhibits a similar trend, namely that this quantity increases from $\overline{E}_{HOMO}$ = 7.01 ± 0.59 eV for 10-mer ssRNA to 8.04 ± 0.13 eV for 10-mer ssDNA and to 8.47 ± 0.61 eV for control 10-mer ssRNA (Table 1), with the lowest value of $\overline{E}_{HOMO}$ for 10-mer ssRNA capable of CT. The results obtained for 5-mer RNA and 5-mer ssDNA follow a similar trend to the one observed for 10-mer ssRNA and 10-mer ssDNA discussed above.

We also analyzed the values of the energy gap, $\Delta E = E_{LUMO} - E_{HOMO}$, for 10-mer RNA, 10-mer DNA, and control 10-mer RNA, accumulated in Table 1. The higher (lower) values of $\Delta E$ are associated with the higher (lower) values of the energy barrier for CT. We found that, for the 10-base length oligonucleotides, $\Delta E$ is the lowest for the 10-mer RNA conformers, compared to those for 10-mer DNA and for control 10-mer RNA. For example, for the most populated conformer I, $\Delta E$ = 0.13 eV for 10-mer RNA, 1.01 eV for 10-mer DNA, and 3.36 eV for control 10-mer RNA (Table 1). A similar trend is observed for the ensemble average values of the energy gap, $\Delta \overline{E}$, which came to 0.56 ± 0.30 eV for 10-mer RNA, 0.79 ± 0.28 eV for 10-mer DNA, and 2.17 ± 1.40 eV for control 10-mer RNA (Table 1). This again confirms a lower barrier for CT along this 10-mer RNA. Thus, the smallest $\Delta \overline{E}$ for 10-mer RNA is in agreement with the experimental observation that only this single-stranded oligonucleotide shows conductivity. While in relatively long 10-base length oligonucleotides, the energy of the HOMO ($E_{HOMO}$) determines CT, in a 5-base oligonucleotide there might be a significant tunneling CT component, where the barrier ($\Delta E$) will play a more significant role.

**Table 1:** Solution conformations of 10-base length oligonucleotides: Shown for each of the most abundant solution conformers I–V and for each 10-mer oligonucleotide sequence indicated are their equilibrium population weight (*w*), end-to-end distance (*X*), number of base stacks ($N_{BS}$), number of base pairs ($N_{BP}$), energy of the HOMO ($E_{HOMO}$) and energy gap ($\Delta E$).

	*w*	*X*, nm	$N_{BS}$	$N_{BP}$	$E_{HOMO}$, eV	$\Delta E$, eV
Sequence CUCCAACAUC						
10-mer RNA I	0.31	3.70	2	0	6.32	0.13
10-mer RNA II	0.21	3.13	5	0	7.47	0.64
10-mer RNA III	0.19	1.91	5	0	7.11	0.73
10-mer RNA IV	0.12	1.46	4	0	7.53	0.88
10-mer RNA V	0.06	2.34	3	1	7.63	1.27
Average	–	2.79 ± 0.96	3.69 ± 1.50	0.07 ± 0.28	7.01 ± 0.59	0.56 ± 0.30
Sequence CTCCAACATC						
10-mer DNA I	0.22	1.79	2	3	7.98	1.01
10-mer DNA II	0.15	1.58	4	1	8.22	0.36
10-mer DNA III	0.12	1.14	3	2	7.88	0.87
10-mer DNA IV	0.12	0.97	6	1	8.06	0.89
10-mer DNA V	0.07	1.52	1	2	8.10	0.68
Average	–	1.49 ± 0.48	3.11 ± 1.75	2.26 ± 1.64	8.04 ± 0.13	0.79 ± 0.28
Sequence CUCCACUCCA						
Control 10-mer RNA I	0.32	1.13	4	0	8.53	3.36
Control 10-mer RNA II	0.21	1.20	5	0	9.19	0.61
Control 10-mer RNA III	0.10	1.19	1	4	8.25	3.64
Control 10-mer RNA IV	0.08	2.81	4	0	7.35	0.81
Control 10-mer RNA V	0.05	2.73	5	0	7.30	0.35
Average	–	1.48 ± 0.67	4.07 ± 1.42	0.67 ± 1.58	8.47 ± 0.61	2.17 ± 1.4

Also shown are the ensemble average quantities (and standard deviations): average end-to-end distance ($\overline{X}$), average number of base stacks ($\overline{N}_{BS}$), average number of base pairs ($\overline{N}_{BP}$), average energy of the HOMO ($\overline{E}_{HOMO}$), and average energy gap ($\Delta \overline{E}$). The average quantities are calculated using the conformations I–V that account for $\sim$85% of the equilibrium population (i.e. $\sum _i w_i$ $=$0.85).

We compared the contributions to the HOMO for entire molecular structures of 10-mer ssRNA, 10-mer ssDNA, and control 10-mer ssRNA from the sugar-phosphate backbone and from the nucleobases. For these sequences, we performed quantum chemistry calculations for the molecular fragments of structures I-III (Table 1) formed only by the backbone (without bases) and by the nucleobases (without a backbone). The results obtained are accumulated in Table S1, which compares the values of $E_{HOMO}$ for the backbone and for the nucleobases. Both for the sugar-phosphate backbone and nucleobases, the average energies $\overline{E}_{HOMO}$ are the lowest for the 10-mer ssRNA (8.04 ± 1.07 eV and -5.68 ± 0.06 eV, respectively (Table S1) as compared with the other two oligonucleotides, for which the values of $\overline{E}_{HOMO}$ are higher, i.e. 8.86± 0.12 eV and 2.48 ± 6.39 eV for 10-mer ssDNA, respectively, and 9.68 ± 0.34 eV and -0.40 ± 8.50 eV for control 10-mer ssRNA, respectively (Table S1). This supports the experimental observation that only the 10-mer ssRNA conducts. Interestingly, the values of $\overline{E}_{HOMO}$ for nucleobases are lower than those for the sugar-phosphate backbone (Table S1), suggesting that CT is mediated mainly by low-energy orbitals delocalized over a significant portion of the molecules comprising the stacked bases in these short oligonucleotides for this moderate bias voltage (and energy) range.

## Conclusion

In summary, we report the first study of single-molecule electrical conductance on single-stranded RNA. By directly coupling the STM-BJ approach with computer simulations and quantum chemistry calculations, here we explored the electronic properties of particular sequences capable of base stacking of 5-mer and 10-bases single-stranded oligonucleotides. These ssRNAs, base-stacked and extended in structure, have conductance values in the ${10^{-3}}G_0$ range (around 230 nS), while single-stranded DNA and a control (nonconducting) ssRNA, both less base-stacked and more compact structures, do not have measurable molecular conductance. Double-stranded dsRNA has a conductance slightly higher than that of the ssRNA counterparts, indicating that increased helicity of extended structures with greater base-stacking favors CT in these short oligonucleotides. Structural insights from the CD spectral lineshapes, measured experimentally and reconstructed theoretically with help from Machine Learning, confirm the importance of more extended helical conformations for CT. In these short sequences, ssRNA adopts an open extended form with more base stacking than ssDNA, while ssDNA forms less base-stacked conformations that tend to close on themselves through more intrastrand base pairing. Consequently, ssDNA is less accessible to bind the electrodes at these single-molecule junctions and at the same time have worse CT pathways. Therefore, more delocalized electronic orbitals exist over a significant portion of the molecule in the more extended ssRNA, explaining the higher conductance. All these multiple pieces of evidence support the idea that molecular conductivity is favored by extended and relatively base stacked oligonucleotides, while more compact and less base-stacked structures have no measurable single-molecule conductance, which agrees with the notion that CT occurs mainly through the orbitals of the stacked bases in this moderate energy range. This could also be compatible with similar effects happening in backbone-mediated CT at higher energies.

## Methods

### Single molecule conductance measurements

All break junction experiments were performed using a Molecular Imaging Pico-STM head connected to a Digital Instruments Nanoscope IIIa controller at room temperature with a 10 nA/V current preamplifier. A LabView (National Instruments) program was used to control the movement of the STM tip using a PCIe-6229 DAQ card (National Instruments) during the measurements. Control measurements were carried out by bringing an atomically sharp Apiezon wax-coated Au tip (Goodfellow) to an Au(111) surface in the presence of a buffer solution before adding oligonucleotides. 15 µL of oligonucleotide samples (nM range) were then injected into the cell. The process of approaching the surface was done with an atomically sharp tip with a constant bias of 20 mV, then retracted at the same rate while the current was recorded. The formation of a molecular junction is indicated by a step or an interruption in the current-distance exponential trace^32^. By accumulating thousands of those traces (4000–5000), we generate the conductance ($G=I/V$) histograms using automated LabVIEW software^18^. The selectivity of curves to build a histogram (number of curves selected by LabVIEW program/total number of curves) is around 3% for each histogram. The LabVIEW program performs a logarithmic binning of the current versus distance traces to create a single-trace histogram and then detects when the conductance steps yield peaks in the histogram that are higher than a certain number of counts (250–300 curves in each dataset). All curves meeting the criteria (exponential shape and a threshold step length) are selected and added to a semi-logarithmic histogram. A Gaussian fitting on top of the logarithmic histogram of the background signal gives us the most probable conductance (1D) for that particular molecular junction. 2D logarithmic conductance-distance histograms can be viewed as an overlapping image of all experimentally obtained conductance-distance traces acquired during the elongation of molecular junction^16,33^. Black dashed lines represents the weighted average of the log(G/$G_0$) values. The relative conductance histograms are obtained from the traces between the blue dashed line in the 2D histogram. We also used an alternative approach based on current-time recordings at a fixed distance^19,20^. After approaching the probe to a tunneling distance with the substrate, the STM feedback was disconnected and the tunneling current was recorded as a function of time. When individual biomolecules spontaneously bridge between probe and substrate electrodes, sudden current steps or ’blinks’ were observed in the current trace (Fig. S2a–c). We record the same conductance signals with different current setpoints, indicating that variations in the tip-to-sample distance are not affecting the molecule conformation or its conductance in this range. Figure S2a, shows that we have measured the same conductance value for RNA sequences with different initial current setpoints (different tip to sample distance). The amplitude of individual current traces can then be transformed into a conductance histogram to calculate the most probable conductance value for that particular junction formation event (Fig. S2d,e).

### Sample preparation

single-stranded RNA and DNA oligonucleotides, 5-base length (CUCCA and CTCCA) and 10-base length (CUCCAAUAUC and CTCCAATATC) were purchased from Biosynthesis Inc. (USA). Some sequences had thiol linkers and C6 spacers on both 5'- and 3'- ends in the same strand for electrode binding (RNA probes). Single-stranded oligonucleotides were received in powder form, which was spun down and then resuspended in 100 mM phosphate buffer (pH 7.4). Tris (2-carboxyethyl) phosphine (TCEP) was used to reduce the disulfide bond in the 5' and 3' positions. TCEP selectively and completely reduces even the most stable water-soluble alkyl disulfides in a wide pH range. This treatment allows producing free thiol groups from the oligonucleotide sequence. Double-stranded sequences were prepared by hybridizing them in a 1:1 ratio with their respective complementary strands (in the nM range) in buffer. Thermal hybridization was carried out by heating the mixture to 80°C in a water bath. After that, the hybridized samples were left to cool at room temperature for several hours. In-situ hybridization was performed by adding the complementary strand (target) to the individual 5 and 10-base-length single-stranded RNA (probe) sequences at room temperature directly into the STM cell (without heating). The incubation period was around 30 min for the entire in situ hybridization process.

### Circular dichroism experiments

Circular dichroism experiments were performed using a JASCO J-1500 CD spectrometer with a 1-mm path length cylindrical quartz cell (250 µL) at 25°C. Before every measurement with oligonucleotide samples, a central blank spectrum (phosphate buffer) was collected and subtracted from the sample data. 40 µL of 150 mM oligonucleotide sample was added to the cell prior to the collection of spectrophotometry data. The final spectra were smoothed by taking an average of 5 scans.

### Computational molecular modeling

#### Structure building

The initial conformations of the single-stranded RNA were obtained using the SimRNA software package^34^. A DNA strand was constructed by replacing 2'-OH group by 2'-H group in ribose rings using VMD^35^ package. Using TIP3P water molecules, each single-stranded oligonucleotide system was solvated up to 15 Å from any of the solute atoms. The number of water molecules for 5-mer and 10-mer RNAs and DNAs included in the octahedral solvation box of volume  95 nm^3^ and  250 nm^3^, respectively, was  2700 and  7400, respectively. *Molecular Dynamics simulations* in explicit water were performed as described in the SI. The numerical output from the MD simulations, i.e. coordinate and energy files, and structure snapshots of each molecule extracted at every 1-ns time interval, were used in data analyses and modeling carried out as described in the SI. The thermodynamic state functions *G*, *H* and *S* were calculated as described in SI. *Quantum chemistry calculations:* The density functional theory (DFT) based calculations were performed using the Gaussian 16 package^36^. Single-point energy calculations were performed using the B3LYP/6-31G(d,p) basis set. We calculated the energies of molecular orbitals $E_{HOMO}$ and the values of energy gap $\Delta E = E_{HOMO} - E_{LUMO}$.

### Resolving oligonucleotide solution conformations

Theoretical calculations of CD spectra for oligonucleotide structures from MD simulations were carried out as described in the SI. The average theoretical CD spectra $\Theta _{th}$ were obtained using the following non-linear regression algorithm, which was already used in a recent publication^30^. *Step 1*: Assign a random weight (i.e. ensemble population) $w_{i}$ to each CD profile $\theta _{i}$ representing the *i*-th conformation in the ensemble of a total of *N* conformations. *Step 2*: Form a weighted superposition, $\Theta _{th}(\lambda ) = w_1\theta _1(\lambda )+w_2\theta _2(\lambda ) + \cdots + w_N\theta _N(\lambda )$ in order to obtain the theoretical spectrum $\Theta _{th}(\lambda )$. *Step 3*: Calculate the mean squared error (MSE), $\text {MSE} = \frac{1}{m}\sum _{j=1}^{m}\left[ \Theta _j(\lambda _j)-\Theta _{th,j}(\lambda _j)\right] ^2$ by comparing the values of experimental CD data point $\Theta _j$ and theoretical prediction $\Theta _{th j}$ for all values of the wavelength $\lambda _j, j = 1, 2, \ldots , m$, (*m* is the total number of data points in the CD spectrum). *Step 4*: Minimize the MSE by varying the populations $w_i$ for all $i = 1, 2, \ldots , N$ using the stochastic gradient descent algorithm. The 5 most important conformations (I, II, III, IV, and V) with the largest statistical weights (equilibrium populations) $\ge$ 0.05 were selected for further analysis and modeling.

### Supplementary Information

Supplementary Information.

Supplementary Movie S1.

Supplementary Movie S2.

Supplementary Movie S3.

## Data Availability

Representative datasets generated and/or analysed during the current study from MD simulations are publicly available in the github repository, https://github.com/jmartes1/ssRNAandDNA2023.
