# Supplementary material for: Charge transport in individual short base stacked single-stranded RNA molecules
Source: Sci Rep. 2023 Nov 13;13:19858. doi: 10.1038/s41598-023-46263-0 (PMC10645971; doi:10.1038/s41598-023-46263-0)
Supplement: Supplementary file 1 — Supplementary Information. [file 41598_2023_46263_MOESM1_ESM.pdf]

SUPPLEMENTARY INFORMATION

Supplementary Methods

Substrate Preparation:

We use an Au(111) single crystal (Goodfellow) as one of the electrodes for depositing thiolated RNA and DNA oligonucleotide samples. Before each experiment, Au single crystal was electropolished in 0.1 M H_2SO_4 (Sigma Aldrich) using two thin gold wire loops as electrodes. A single Au crystal was placed on top of one gold wire loop (working electrode) and connected to the DC power supply. The other gold wire loop (counter electrode) was placed closer to the single crystal (preferably less than 0.5 cm) and connected to the same DC supply. We used the DC power supply to achieve a voltage of 10 V and a current of about 1 A. The current was applied for 20-30 sec until we observed that the Au single crystal had oxidized to form an orange layer on top. The oxidized Au single crystal was then rinsed in Milli-Q water multiple times. The rinsed Au single crystal was placed into 1M HCl (Sigma Aldrich) solution for 2 minutes and rinsed again in Milli-Q water until we observed the shiny pure gold color back again. Finally, the clean Au single crystal was air-dried, before being annealed using a butane flame for about 1 minute to make the surface atomically flat and ready for the experiment.The STM tip was prepared by cutting a 0.25 mm gold wire (Goodfellow) sharply at an angle. We covered the STM tips with Apiezon wax (Apiezon) to isolate them from any leakage current from the ions in the medium to prevent their integration with molecular conductance.

Melting temperature calculations for single-stranded RNA oligonucleotide :

To measure the melting temperature of ssRNA oligonucleotide strands, we use formula: $T_m = 2(A+U)+4(C+G)-7$, where T_m is the melting temperature (in °C), A is the number of adenosine nucleotides, U is the number of uracil nucleotides, C is the number of cytidine nucleotides, G is the number of guanosine nucleotides in the sequence, and -7 is correction factor accounting for in a solution.

Buffer preparation:

We resuspend the oligos in a weak buffer (phosphate) with a pH of 7.4. NaH_2PO_4 and Na_2HPO_4 were purchased from Sigma-Aldrich to prepare 100 mM phosphate buffer with a pH of 7.4 with Milli-Q water. The buffer solution was then filtered with 20 nm pore size filters (Whatman Anotop 25 plus) before using in the experiment.

Cleaning solution preparation:

Piranha solution was used to clean all glassware and Teflon STM cells. The solution was prepared by adding 98% H_2SO_4 and 30% H_2O_2 in a 3:1 v/v ratio. Other parts of the STM cell and parts of the microscope were cleaned with CH_3CHO (HPLC plus) and CH_3CH_2OH (pure) purchased from Sigma-Aldrich.

Labview program:

A customized LabView (National Instruments) program was used to control the movement of the STM tip during the measurements[1]. This program performs a logarithmic binning of each recorded current-distance trace and then selects those whose residual values meet the specified criteria. All filtered curves were added together to obtain a semilogarithmic conductance histogram, which revealed the most probable conductance for a specific molecular junction. Our histograms typically included hundreds of curves (only curves with plateaus or nonexponential features) from datasets comprising thousands of experiments overall. These numbers are indicated in each figure caption.

Molecular Dynamics simulations:

Each oligonucleotide system (oligonucleotide plus counterions plus water molecules) was energy-minimized to eliminate possible sterical clashes for 10,000 steps of the steepest descent algorithm (water molecules and counterions), and then for 5,000 steps of the conjugate gradient with 50 kcal/mol restraint placed on the solute atoms. Next, each oligonucleotide system was heated from 0 K to 300 K over 50 ps time. Solvent molecules were relaxed using short MD simulations (250 ps) with 30 kcal/mol restraint on the solute atoms at T = 300 K temperature. The equilibration step involved 100 ps of restrained MD simulations with 0.05 kcal/mol restraint placed on solute atoms. The MD simulations in explicit solvent (water) for all oligonucleotide systems were carried out as reported in prior studies[2–4] using the periodic boundary conditions. Finally, the 2-μs long unrestrained equilibrium MD simulations (production runs) for all single-stranded oligonucleotide systems were performed in the NPT ensemble using the CUDA version of pmemd[5] in conjunction with the GPU accelerated version[6] of AMBER 20[7]. The Particle Mesh Ewald method was utilized to calculate the long-range electrostatics with the cutoff distance of 10 Å. SHAKE algorithm was used to treat the hydrogen atoms. All the equilibrium MD simulations for all oligonucleotide systems were carried out at T = 300 K temperature using Δt = 2 fs integration step. In these simulations, the 1-atm constant pressure was maintained using a Berendsen barostat with a 1-ps time constant[8]. The 300 K constant temperature was maintained using a Berendsen thermostat with a 4-ps time constant.

Analysis of MD simulations:

The end-to-end distance X was calculated as the distance between the P-atoms of the first and last nucleotides. The radius of gyration R_g was calculated using the coordinates of all atoms and the formula, $R_g = \sum_q^Q m_q \mathbf{r}_q^2 / \sum_q^Q m_q$, where m_q is the mass of atom q and $\mathbf{r}_q$ is the position of atom q, relative to the center-of-mass of the molecule. The Solvent Accessible Surface Area (SASA) was estimated using the LCPO algorithm[9] implemented in the CPPTRAJ module[10] in AmberTools20[7]. The numbers of base pairs N_{BP} and base stacks N_{BS} were calculated using Barnaba software[11]. The structure schematic for the calculation of N_{BP} and N_{BS} is shown in Fig. S6. Briefly, bases were classified as stacked if $|z_{kj}|$ and $|z_{jk}| > 2$ Å, ρ_{kj} or $\rho_{jk} < 2.5$ Å, and $|\alpha_{kj}| < 40°$. Here, $\rho_{kj} = \sqrt{x_{kj}^2 + y_{kj}^2}$, where the x- and y-axes are in the plane of the base (x_{kj} and y_{kj} are the distances between the centers-of-mass of the two bases along the x- and y-axes, respectively) and the z-axis is normal to the xy-plane (z_{kj} is the distance between the centers of mass of the two bases), and α_{kj} is the angle between the normal vectors of the two bases (Fig. S6)[11]. All the non-stacked bases are considered to be base-paired if $|\alpha_{kj}| < 60°$ and there exists at least one hydrogen bond (H-bond) between the k-th and j-th bases (Fig. S6). We assume that the H-bond D–H...A between the hydrogen donor atom (D) and acceptor atom (A) is formed if the donor–acceptor distance d_{DA} is less than 3.3 Å cutoff and the bond angle is larger than the 140° cutoff[11]. To avoid analyzing similar conformations, in structure selection we used the eRMSD measure of structural similarity[12] implemented in the Barnaba software[11]. Briefly, the eRMSD is a contact map-based distance metric, with the addition of a number of features that make it suitable for the comparison of structures of nucleic acids. We used eRMSD to pre-screen the output from MD simulations for 5-mer and 10-mer oligonucleotide systems and to discard similar structures from subsequent data analysis. To distinguish between the closed and open conformations of the 10-mer RNA, 10-mer DNA and control 10-mer RNA, we carried out extensive structure analysis. We found that the end-to-end distance X and the number of hydrogen bonds N_{HB} can be used to distinguish between the 'open' and 'closed' conformational states. All three oligonucleotides were found to adopt a collapsed structure stabilized by the maximum number of hydrogen bonds (i.e. N_{HB} = 10 for 10-mer DNA, and N_{HB} = 16 for 10-mer RNA and 10-mer control RNA) only when $X < 1.9$ nm. Based on these observations, we classified all the structure snapshots as the collapsed conformations ('closed' state) and extended conformations ('open' state) if $X < 1.9$ nm and $X > 1.9$ nm, respectively.

Theoretical calculation of CD spectra:

To select structurally different conformations, we use the eRMSD measure of structure similarity[11], which varies between 0.4 and 2.0 for all oligonucleotides. These ranges are practically the same as was observed for MD simulations of different sequences[12]. For each oligonucleotide, we selected structures that differ in their eRMSD values by ΔeRMSD > 0.002. These structures were then used to calculate the theoretical CD spectral profiles for the oligonucleotide molecules. Next, for each conformer and for each model system (5-mer RNA, 5-mer DNA, 10-mer RNA, 10-mer DNA and control 10-mer RNA), we calculated theoretical Circular Dichroism (CD) spectral profiles using the matrix method[13–15]. In this method, one calculates the interactions between the various electronic excitations to determine the values of rotational strength. This yields a CD profile, i.e. a set of values for the rotational strength for each electronic transition as a function of the wavelength λ, $\theta(\lambda)$[16]. The methodology for the theoretical calculation of a CD spectrum is implemented in DichroCalc software[16,17].

Thermodynamic State Functions:

The Gibbs free energy G, enthalpy H and entropy S of oligonucleotide were calculated for each j-th conformer observed in equilibrium MD simulations, where $j = 1,2,...,N$ (N is the total number of conformations for oligonucleotide). To calculate G, H, and S, we utilized the Molecular Mechanics/Generalized Born Surface Area (MM/GBSA) method[18] implemented in the MMPBSA.py program[19]. The enthalpy of a state H is estimated as $H = E_{int} + E_C + E_{vdW} + E_p + E_{np}$. Here, the first three terms are standard Molecular Mechanics potentials, which describe the bond length potential, bond angle potential, and dihedral angle potential (included in E_{int}), electrostatic interaction potential (E_C) and van der Waals interaction potential (E_{vdW}). In this equation, E_p and E_{np} are the polar and non-polar contributions to the solvation free energies, respectively; E_p is obtained by using the generalized Born (GB) model, and E_{np} is estimated using the solvent accessible surface area (SASA). The entropy of a state S is estimated by the normal-mode analysis of the vibrational frequencies[20].

References

1. Hihath, J. & Tao, N. Rapid measurement of single-molecule conductance. *Nanotechnology* **19**, 265204 (2008).
2. Bottaro, S., Bussi, G., Kennedy, S. D., Turner, D. H. & Lindorff-Larsen, K. Conformational ensembles of RNA oligonucleotides from integrating NMR and molecular simulations. *Sci. Adv.* **4**, eaar8521 (2018).
3. Harikrishna, S. & Pradeepkumar, P. Probing the binding interactions between chemically modified siRNAs and human argonaute 2 using microsecond molecular dynamics simulations. *J. Chem. Inf. Model.* **57**, 883–896 (2017).
4. Krepl, M. *et al.* Can we execute stable microsecond-scale atomistic simulations of protein–RNA complexes? *J. Chem. Theory Comput.* **11**, 1220–1243 (2015).
5. Darden, T., York, D. & Pedersen, L. Particle mesh Ewald: An N log (N) method for Ewald sums in large systems. *Chem. Phys.* **98**, 10089–10092 (1993).
6. Salomon-Ferrer, R., Gotz, A. W., Poole, D., Le Grand, S. & Walker, R. C. Routine microsecond molecular dynamics simulations with AMBER on GPUs. 2. Explicit solvent particle mesh Ewald. *J. Chem. Theory Comput.* **9**, 3878–3888 (2013).
7. Case, D. *et al.* *AMBER 2020* (University of California, San Francisco, 2020).
8. Berendsen, H. J., Postma, J. v., Van Gunsteren, W. F., DiNola, A. & Haak, J. R. Molecular dynamics with coupling to an external bath. *Chem. Phys.* **81**, 3684–3690 (1984).
9. Weiser, J., Shenkin, P. S. & Still, W. C. Approximate atomic surfaces from linear combinations of pairwise overlaps (LCPO). *J. Comput. Chem.* **20**, 217–230 (1999).
10. Roe, D. R. & Cheatham III, T. E. PTRAJ and CPPTRAJ: software for processing and analysis of molecular dynamics trajectory data. *J. Chem. Theory Comput.* **9**, 3084–3095 (2013).
11. Bottaro, S. *et al.* Barnaba: software for analysis of nucleic acid structures and trajectories. *RNA* **25**, 219–231 (2019).
12. Bottaro, S., Di Palma, F. & Bussi, G. The role of nucleobase interactions in RNA structure and dynamics. *Nucleic Acids Res.* **42**, 13306–13314 (2014).
13. Johnson, W. C. Determination of the conformation of nucleic acids by electronic CD. In *Circular dichroism and the conformational analysis of biomolecules*, 433–468 (Springer, 1996).
14. Micsonai, A. *et al.* Accurate secondary structure prediction and fold recognition for circular dichroism spectroscopy. *Proc. Natl. Acad. Sci. U.S.A.* **112**, E3095–E3103 (2015).
15. Chin, S. L. *et al.* Combined molecular dynamics simulations and experimental studies of the structure and dynamics of poly-amido-saccharides. *J. Am. Chem. Soc.* **138**, 6532–6540 (2016).
16. Bulheller, B. M., Rodger, A. & Hirst, J. D. Circular and linear dichroism of proteins. *Phys. Chem. Chem. Phys.* **9**, 2020–2035 (2007).
17. Bulheller, B. M. & Hirst, J. D. Dichrocalc—circular and linear dichroism online. *Bioinformatics* **25**, 539–540 (2009).
18. Hou, T., Wang, J., Li, Y. & Wang, W. Assessing the performance of the MM/PBSA and MM/GBSA methods. 1. the accuracy of binding free energy calculations based on molecular dynamics simulations. *J. Chem. Inf. Model.* **51**, 69–82 (2011).
19. Miller III, B. R. *et al.* MMPBSA.py: an efficient program for end-state free energy calculations. *J. Chem. Theory Comput.* **8**, 3314–3321 (2012).
20. McQuarrie, D. A. *Statistical mechanics* (Sterling Publishing Company, 2000).
21. Humphrey, W., Dalke, A. & Schulten, K. VMD: visual molecular dynamics. *J. Mol. Graph.* **14**, 33–38 (1996).
22. Sebechlebská, T. *et al.* Additive transport in dna molecular circuits. *J. Mater. Chem. C* **10**, 12022–12031 (2022).

Table S1. Energies of the frontier molecular orbitals for the sugar-phosphate backbone and nucleobases for three 10-mer oligonucleotides: Shown for the sugar-phosphate backbone and nucleobases of each principal solution conformer I-III are the equilibrium population (w), energy of the HOMO (E_{HOMO}), and energy gap (ΔE). Also shown are the ensemble average quantities (and standard deviations): average energy of the HOMO ($\overline{E}_{HOMO}$), and average energy gap ($\Delta\overline{E}$) calculated using the conformations I-V (see Table **??** in the main text) that account for $\sim$85% of the equilibrium population (i.e. $\sum_i w_i = 0.85$). The values obtained for the backbone and nucleobases are separated by a slash.

	w	E_{HOMO}, eV	ΔE, eV
10-mer RNA I	0.31	6.82/-5.73	2.37/2.81
10-mer RNA II	0.21	9.03/-5.71	0.86/2.45
10-mer RNA III	0.19	8.92/-5.58	0.36/2.37
average	-	8.04 ± 1.07/-5.68 ± 0.06	1.39 ± 0.89/2.59 ± 0.20
10-mer DNA I	0.22	8.84/1.20	0.43/0.50
10-mer DNA II	0.15	9.02/11.09	1.23/3.79
10-mer DNA III	0.12	8.70/-5.95	1.79/0.55
average	-	8.86 ± 0.12/2.48 ± 6.39	1.01 ± 0.56/1.52 ± 1.51
control 10-mer RNA I	0.32	9.55/-6.05	1.59/4.28
control 10-mer RNA II	0.21	10.12/11.61	2.53/0.28
control 10-mer RNA III	0.10	9.15/-7.51	2.15/2.54
average	-	9.68 ± 0.34/-0.40 ± 8.50	1.99 ± 0.43/2.67 ± 1.80

Supplementary Movie 1: Conformational dynamics of 10-mer DNA: The movie shows conformational fluctuations in 10-mer DNA as observed in the all-atom MD simulations. The MD simulation runs were carried out in explicit water with 0.15 M NaCl. The dynamics of transitions between open and closed states is also shown using the example of one of the most representative parameters: end-to-end distance X. The structure is shown in Twister representation (blue line running through backbone) and in PaperChain representation (for nucleic bases). Shown is a 2-μs long MD simulation run. The length of the movie is 16 s. The movie is played 8×10^6 times slower than the computational experiment.

Supplementary Movie 2: Conformational dynamics of 10-mer RNA: The movie shows conformational fluctuations in 10-mer RNA as observed in the all-atom MD simulations. The MD simulation runs were carried out in explicit water with 0.15 M NaCl. The dynamics of transitions between open and closed states is also shown using the example of one of the most representative parameters: end-to-end distance X. The structure is shown in Twister representation (blue line running through backbone) and in PaperChain representation (for nucleic bases). Shown is a 2-μs long MD simulation run. The length of the movie is 16 s. The movie is played 8×10^6 times slower than the computational experiment.

Supplementary Movie 3: Conformational dynamics of control 10-mer RNA: The movie shows conformational fluctuations in 10-mer control RNA as observed in the all-atom MD simulations. The MD simulation runs were carried out in explicit water with 0.15 M NaCl. The dynamics of transitions between open and closed states is also shown using the example of one of the most representative parameters: end-to-end distance X. The structure is shown in Twister representation (the blue line running through the backbone) and in the PaperChain representation (for nucleic bases). Shown is a 2-μs long MD simulation run. The length of the movie is 16 s. The movie is played 8×10^6 times slower than the computational experiment.

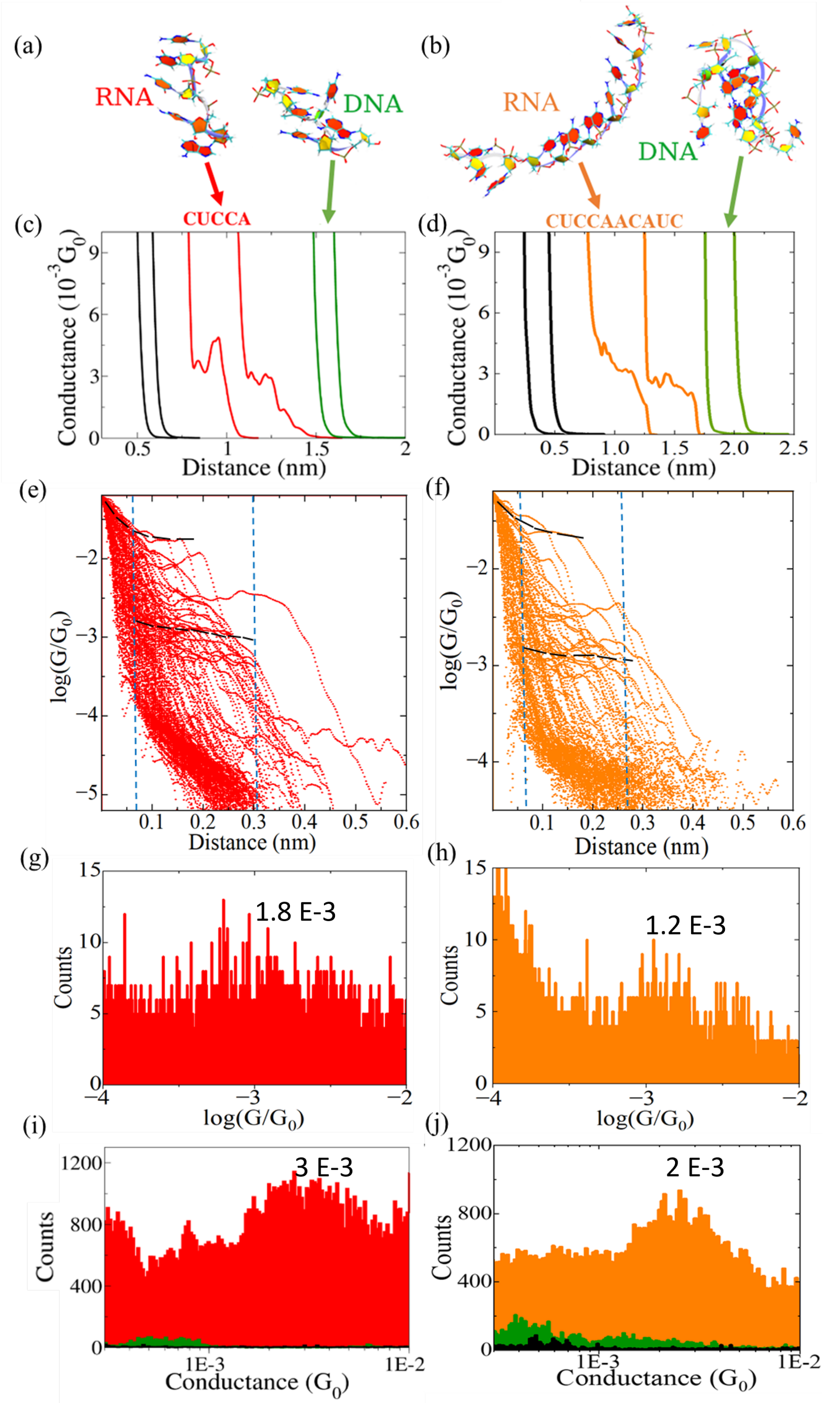

Figure S1. **Single-molecule electrical measurements of single-stranded oligonucleotide sequences:** Molecular Dynamics snapshots of the most representative conformations for (a) 5-mer RNA and DNA and (b) 10-mer RNA and DNA shown in Twister representation (blue line going through the backbone) and in PaperChain representation (for nucleic bases). All the rings are colored by pucker, using the Cremer-Pople pucker amplitude[21]. (c) Conductance vs. distance curve examples (black for phosphate buffer blank, red for ssRNA, and green for ssDNA) for 5-mer sequences. (d)Curves for 10-mer sequences (black for buffer, orange for RNA, green for DNA). Experimental 2D logarithmic conductance–distance (log(G/G_0)– distance) traces for (e) 5-mer RNA (red) and (f) 10-mer RNA (orange). The relative conductance histograms for (g) 5-mer RNA and (h) 10-mer RNA are obtained from conductance traces between the blue dashed lines, the master curves (indicating most probable conductance)[22] are denoted by black dashed lines. (i) 1D logarithmic conductance histogram for 5-mer RNA and (j) 10-mer RNA showing the average conductance around $3\times10^{-3}G_0$ ($G_0 = 2e^2/h = 7.75\times10^{-5}$ A/V is the quantum of conductance). The background signals from a control phosphate buffer experiment are shown in black, RNA data are in red and orange, and DNA signals are in green. Experiments were performed with a constant 20 mV applied bias. Histograms include around 300 curves out of a total around 5000 per experiment.

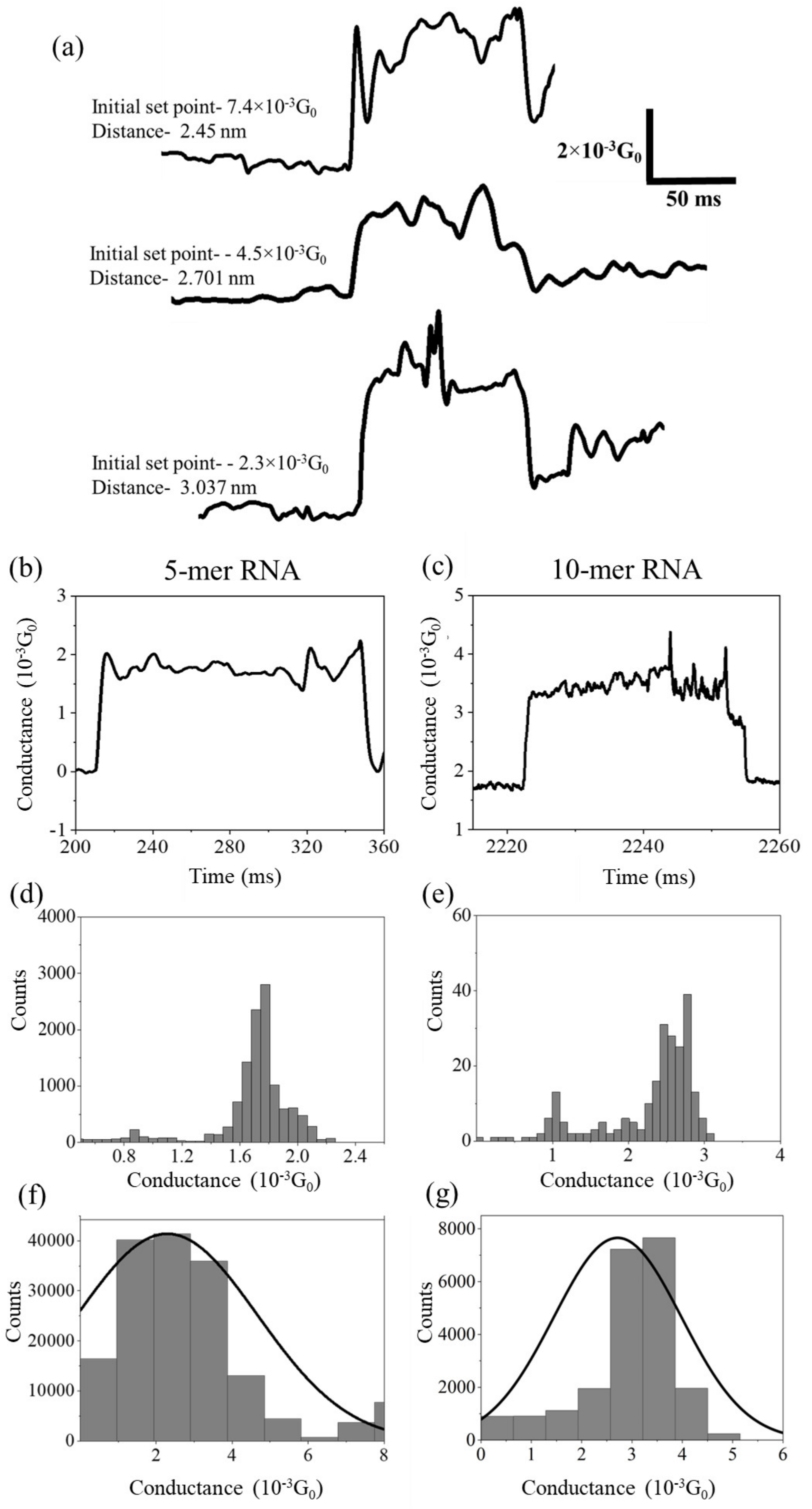

Figure S2. **Results of blinking experiment:** (a) Sample current-distance traces at different current setpoints (different tip to sample distance) for ssRNA sequences. Spontaneous formation of single-molecule 5-mer RNA junctions and 10-mer RNA junctions can be detected by inspecting the current versus time (I vs. t) traces in panels (b) and (c), respectively. Conductance histogram for 5-mer RNA in panel (d) and for 10-mer RNA in panel (e) showing the conductance peaks corresponding to the $I-t$ trace displayed in panel (b) (for 5-mer RNA) and in panel (c) (for 10-mer RNA), respectively. Overall conductance histograms were constructed by adding 20 $I-t$ traces for 5-mer RNA in panel (f) and for 10-mer RNA in panel (g) ($G_0 = 2e^2/h = 7.75 \times 10^{-5}$ A/V is the quantum of conductance) .

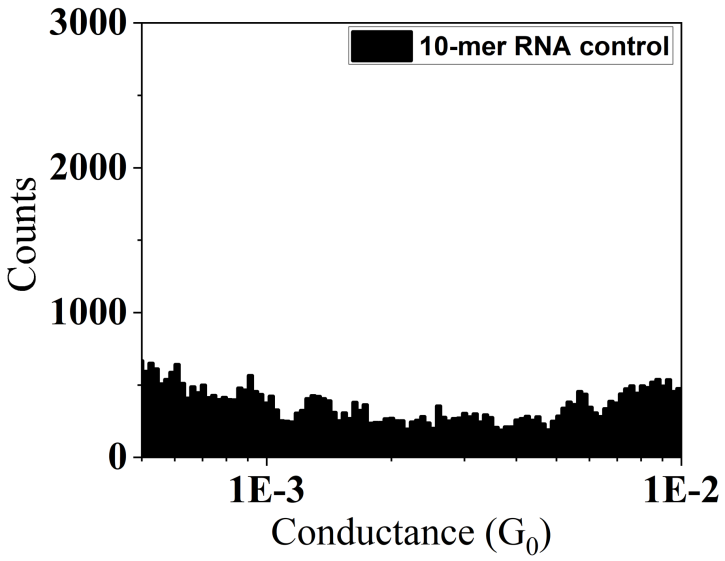

Figure S3. Conductance measurements for control 10-mer RNA: Shown is the conductance histogram in the log-linear representation constructed for control 10-mer ssRNA ($G_0 = 2e^2/h = 7.75 \times 10^{-5}$ A/V is the quantum of conductance).

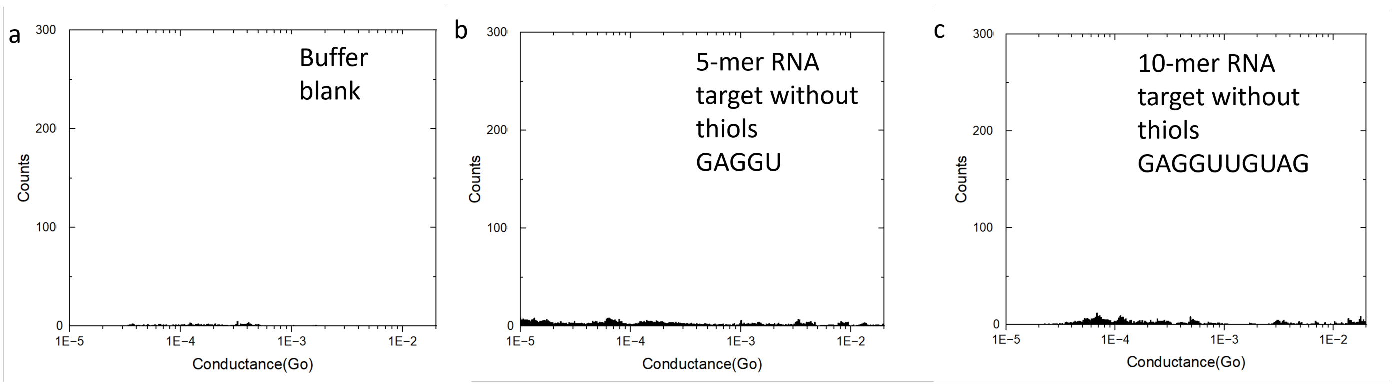

Figure S4. Conductance measurements for control experiments in buffer and RNA targets without thiols: Shown are the conductance histogram in the log-linear representation constructed for (a) a phosphate buffer experiment without biomolecules, (b) the target 5-mer ssRNA sequence indicated without thiols, and (c) the target 10-mer ssRNA sequence indicated also without binding groups. ($G_0 = 2e^2/h = 7.75 \times 10^{-5}$ A/V is the quantum of conductance).

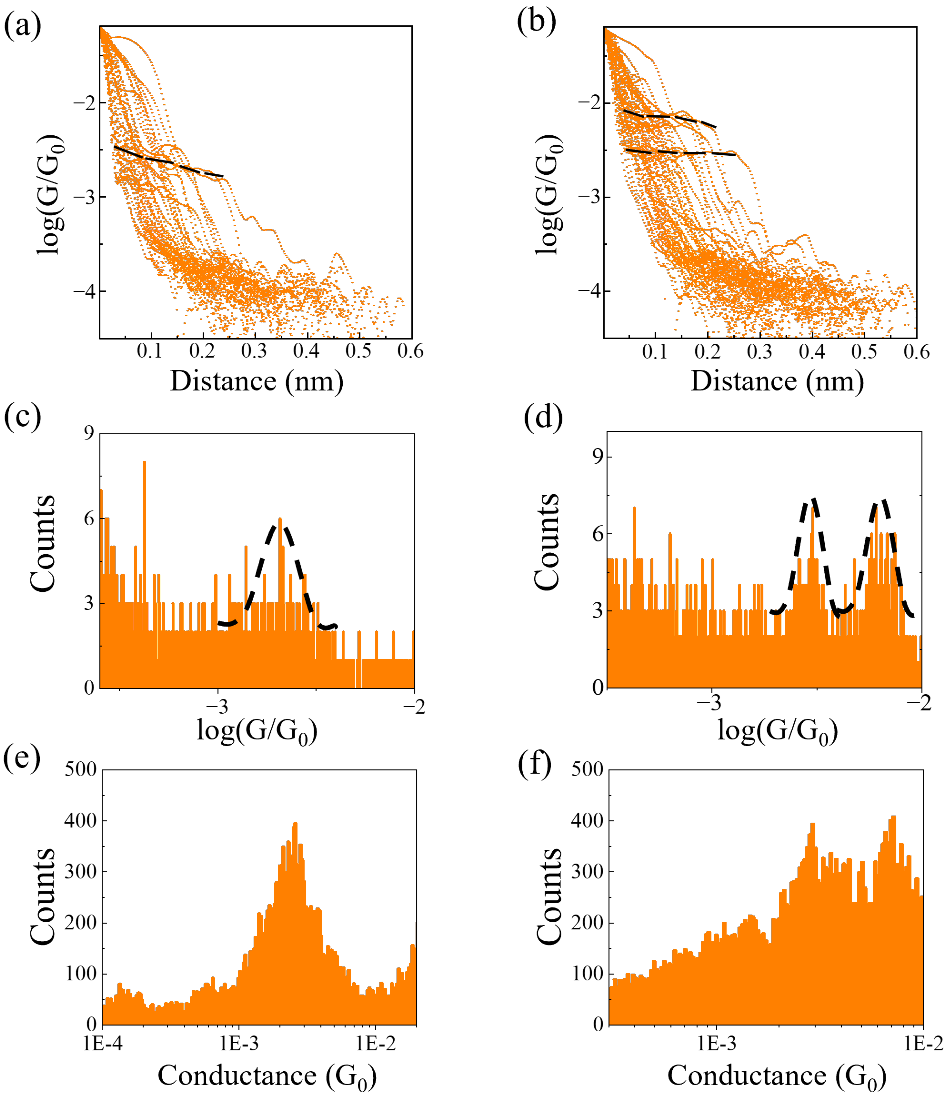

Figure S5. Conductance measurements for *in situ* hybridization of 10 mer RNA : Experimental 2D logarithmic conductance–distance (log(G/G_0)– distance) traces for (a) single-stranded 10-mer RNA before and (b) 10-mer RNA after *in situ* hybridization showing two steps in two different conductance regions. The relative conductance histograms for (c) 10-mer single-stranded RNA and (d) 10-mer RNA after hybridization are obtained from conductance traces between the blue dashed lines, and master curves (indicating most probable conductance) are denoted by black dashed lines. (e) Example 1D conductance traces for 10-mer RNA before hybridization (f) 10-mer RNA after hybridization with two different steps ($G_0 = 2e^2/h = 7.75 \times 10^{-5}$ A/V is the quantum of conductance). Experiments were performed in 100 mM phosphate buffer (pH-7.4) with a constant 20 mV bias. Histograms include around 250 curves of 5000 datasets overall.

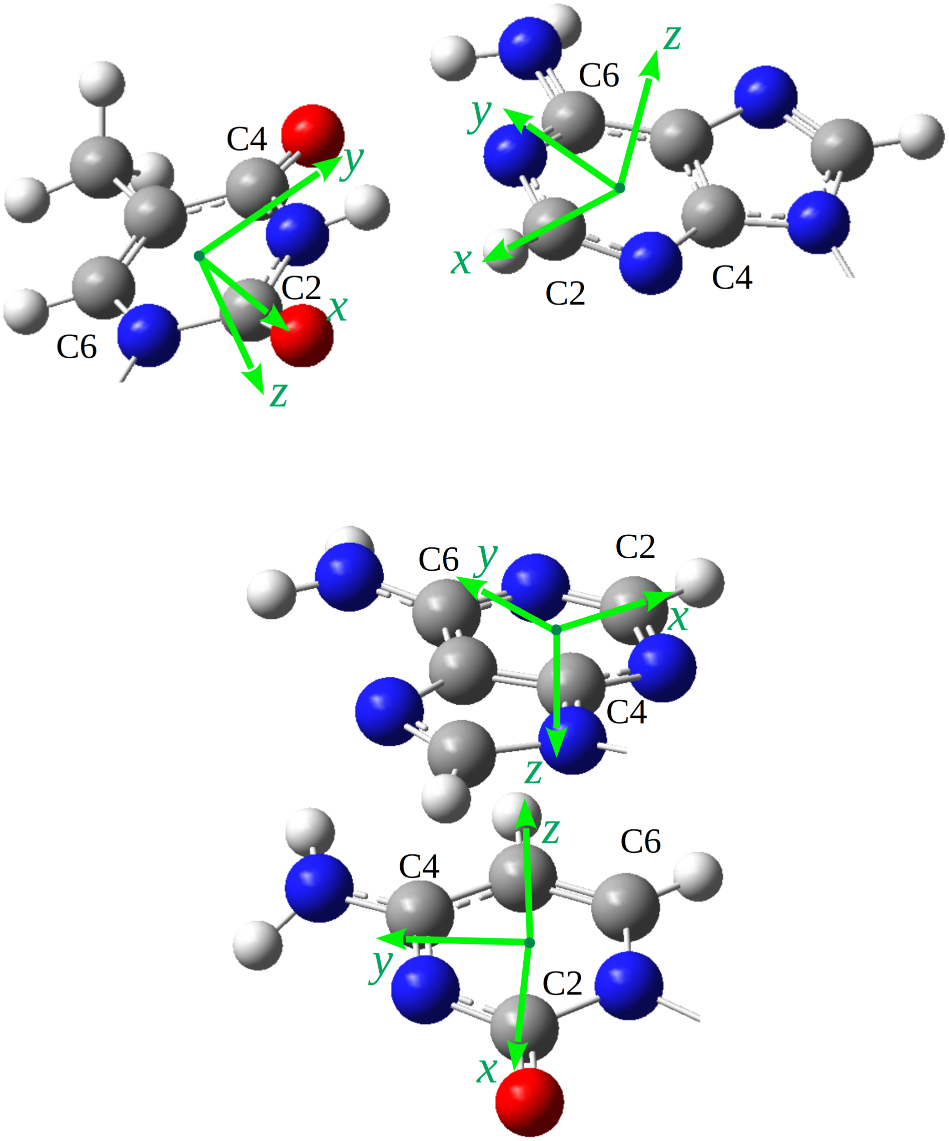

Figure S6. **Base pairing and base stacking interactions:** (a) Local coordinate systems for purines and pyrimidines are used to describe the base pairing interactions. The center of the base ring atoms C2, C4, and C6 represents the origin of the local coordinate system as shown. The *x*- and *y*-axes lie in the plane of the base while the *z*-axis lies normal to the *xy*-plane. The *x*-axis is pointed in the C2-atom direction, and the *y*-axis is pointed toward the C4-atom (for C and U) or toward the C6-atom (for A and G). The two bases are forming a base pair via hydrogen bonds represented as dashed black lines. (b) Local coordinate system used for describing the base stacking interactions. The carbon, oxygen, nitrogen, and hydrogen atoms are shown in grey, red, blue, and white colors, respectively.

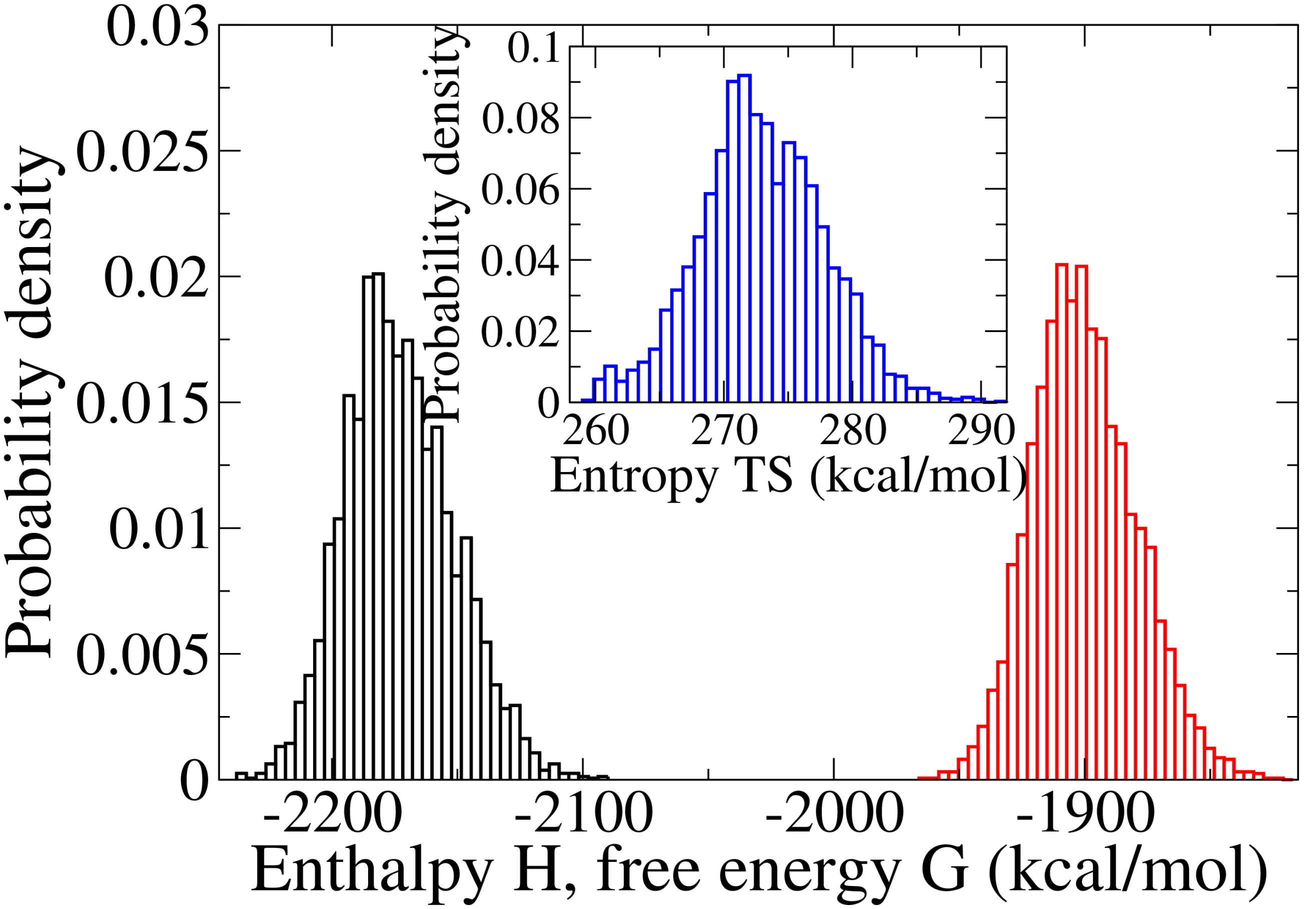

Figure S7. Histograms of thermodynamic state functions for 10-mer RNA: Shown are the histogram-based estimates of the normalized probability density functions (probability distributions) for the values of enthalpy H (red bars), Gibbs free energy G (black bars), and entropy S (blue bars; in *the inset*) for an entire statistical ensemble of conformations of 10-mer RNA. These quantities are obtained based on numerical output from the all-atom MD simulations for the 10-mer RNA at $T = 300$ K temperature (see Methods in main text).
